# Mycobacterium tuberculosis Reactivates HIV-1 via Exosome-Mediated Resetting of Cellular Redox Potential and Bioenergetics

**DOI:** 10.1128/mBio.03293-19

**Published:** 2020-03-03

**Authors:** Priyanka Tyagi, Virender Kumar Pal, Ragini Agrawal, Shalini Singh, Sandhya Srinivasan, Amit Singh

**Affiliations:** a Department of Microbiology and Cell Biology, Centre for Infectious Disease Research, Indian Institute of Science (IISc), Bangalore, India; b International Centre for Genetic Engineering and Biotechnology (ICGEB), New Delhi, India; c Vproteomics, New Delhi, India; Columbia University/HHMI

**Keywords:** glutathione, redox potential, extracellular acidification rate, oxidative phosphorylation, roGFP

## Abstract

The synergy between Mycobacterium tuberculosis and human immunodeficiency virus-1 (HIV-1) interferes with therapy and facilitates the pathogenesis of both human pathogens. Fundamental mechanisms by which M. tuberculosis exacerbates HIV-1 infection are not clear. Here, we show that exosomes secreted by macrophages infected with M. tuberculosis, including drug-resistant clinical strains, reactivated HIV-1 by inducing oxidative stress. Mechanistically, M. tuberculosis-specific exosomes realigned mitochondrial and nonmitochondrial oxygen consumption rates (OCR) and modulated the expression of host genes mediating oxidative stress response, inflammation, and HIV-1 transactivation. Proteomics analyses revealed the enrichment of several host factors (e.g., HIF-1α, galectins, and Hsp90) known to promote HIV-1 reactivation in M. tuberculosis-specific exosomes. Treatment with a known antioxidant—N-acetyl cysteine (NAC)—or with inhibitors of host factors—galectins and Hsp90—attenuated HIV-1 reactivation by M. tuberculosis*-*specific exosomes. Our findings uncover new paradigms for understanding the redox and bioenergetics bases of HIV-M. tuberculosis coinfection, which will enable the design of effective therapeutic strategies.

## Abstract (precis)

Globally, individuals coinfected with the AIDS virus (HIV-1) and with M. tuberculosis (causative agent of tuberculosis [TB]) pose major obstacles in the clinical management of both diseases. At the heart of this issue is the apparent synergy between the two human pathogens. On the one hand, mechanisms induced by HIV-1 for reactivation of TB in AIDS patients are well characterized. On the other hand, while clinical findings clearly identified TB as a risk factor for HIV-1 reactivation and associated mortality, basic mechanisms by which M. tuberculosis exacerbates HIV-1 replication and infection remain poorly characterized. The significance of our research is in identifying the role of fundamental mechanisms such as redox and energy metabolism in catalyzing HIV-M. tuberculosis synergy. The quantification of redox and respiratory parameters affected by M. tuberculosis in stimulating HIV-1 will greatly enhance our understanding of HIV-M. tuberculosis coinfection, leading to a wider impact on the biomedical research community and creating new translational opportunities.

## INTRODUCTION

Tuberculosis (TB) and human immunodeficiency virus/AIDS (HIV/AIDS) jointly represent the major burden of infectious diseases in humans worldwide. HIV-1/Mycobacterium tuberculosis-coinfected patients exhibit rapid progression to AIDS and reduced survival kinetics (1). Moreover, the risk of acquiring active TB infection increases from 10% in a lifetime to 10% per year in the case of HIV-1-infected patients (2). In 2015 alone, WHO estimated that almost 11% of 10.4 million TB patients were also infected with HIV-1 and that one in every three deaths among HIV-1-infected individuals was due to TB (http://www.who.int/hiv/topics/tb/about_tb/en/). Epidemiological studies have clearly indicated that these human pathogens interact to accelerate disease severity and increase death rates (2).

Since infection with HIV-1 significantly increases the risk of TB reactivation in individuals latently infected with M. tuberculosis (3), a great majority of studies have focused on revealing how the virus disorganizes TB granuloma (4), impairs phagosomal killing (5), and alters T-cell-based immunity to exacerbate M. tuberculosis pathogenesis (6). In contrast, whether M. tuberculosis influences exit of HIV-1 from latency and its reentry into a productive life cycle remains poorly studied. Because HIV-1 can persist in a latent state for decades and can then reactivate to cause immunodeficiency, our particular interest is that of understanding the mechanism, if any, underlying M. tuberculosis induced reactivation of HIV-1 from latency. A growing body of evidences suggests that infection with M. tuberculosis or with an M. tuberculosis component(s) (lipids and secretory proteins) promotes HIV-1 replication by regulating processes such as inflammation, major histocompatibility complex class II (MHC-II) processing, signaling by Toll-like receptors (TLRs), CXC chemokine subfamily 4 (CXCR4)/CCR5 expression, production of proinflammatory cytokines/chemokines, and activation of transcriptional regulators (NF-κB, NFAT [nuclear factor of activated T cells]) of the long-terminal repeats (LTRs) of HIV (7–13). The accumulation of contradictory pieces of evidence showing inhibition of HIV-1 replication by M. tuberculosis complicates our understanding of how the two human pathogens interact at the molecular level (14, 15). Despite this, research specifically addressing how M. tuberculosis modulates HIV latency and reactivation is quite scarce. In this context, production of reactive oxygen species (ROS) and modulation of central metabolism are considered to be among the main mechanisms regulating HIV-1 replication, immune dysfunction, and accelerated progression to AIDS (16). Deeper studies in this direction have revealed an important role for a major cellular antioxidant, glutathione (GSH) (17). Low GSH levels in HIV patients have been shown to induce provirus transcription by activation of NF-κB, apoptosis, and depletion of CD4^+^ T cells (18). Consequently, replenishment of GSH is considered to represent a potential supplement to highly active antiretroviral therapy (HAART) (19).

Previously, we reported that subtle changes in the redox potential of GSH (*E_GSH_*) modulate the replication cycle of HIV-1 (20). We discovered that a marginal increase in the levels of intracellular *E_GSH_* (25 mV) is sufficient to reactivate HIV-1, raising the potential of targeting of HIV-1 latency by the modulators of cellular GSH homeostasis (20). Interestingly, levels of markers of oxidative stress such as ROS/reactive nitrogen species (RNS) and lipid peroxidation were found to be elevated in patients with active TB (21). Specifically, serum/cellular GSH was either depleted or oxidized in human TB patients and in the lungs of M. tuberculosis-infected guinea pigs (21, 22). Treatment with a GSH precursor, N-acetyl cysteine (NAC), reversed oxidative stress to reduce bacterial survival and tissue damage in guinea pigs (22). Additionally, M. tuberculosis infection has recently been shown to influence carbon flux through glycolysis and the tricarboxylic acid (TCA) cycle in infected macrophages (23). This, along with the recognized role of GSH homeostasis and glycolysis in HIV infection, indicates that the two pathogens might synergize via affecting redox and energy metabolism of the host. We explored this connection and investigated whether M. tuberculosis coordinates HIV-1 reactivation by affecting *E_GSH_* and bioenergetics. We showed that M. tuberculosis exploits the exosome-based mechanisms to reactivate latent HIV-1. Mechanistically, M. tuberculosis*-*specific exosomes alter gene expression, redox metabolism, and bioenergetics of latent cells to promote HIV-1 reactivation. Proteomic analysis of exosomes identified host pathways known to reactivate HIV-1 by perturbing redox metabolism, inflammation, and immune response.

## RESULTS

### M. tuberculosis infection induces oxidative stress in bystander macrophages.

**FIG 1:**
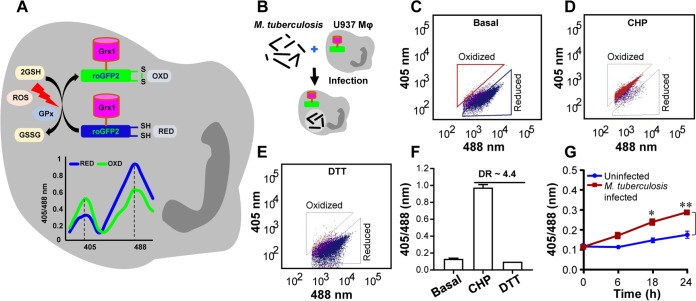
M. tuberculosis induces oxidative shift in *E_GSH_* of U937 macrophages (Mφ). (A) Schematic representation of Grx1-roGFP2 oxidation and reduction in response to ROS inside a mammalian cell stably expressing the biosensor. “GPx” denotes GSH-dependent glutathione peroxidase. The graph represents the ratiometric response (405/488) of Grx1-roGFP2 upon exposure to oxidative (OXD) or reductive (RED) stress. Oxidative stress increases fluorescence at 405-nm excitation and decreases fluorescence at 488 nm with constant emission of 510 nm, whereas an opposite response is induced by reductive stress. (B) PMA-differentiated U937 Mφ stably expressing Grx1-roGFP2 in the cytosol were infected with M. tuberculosis H37Rv at an MOI of 10. (C to E) At indicated time points, ratiometric sensor response was measured using flow cytometry. Dot plots show the ratiometric shift in biosensor response seen with (C) untreated U937 (basal) and upon treatment of U937 with (D) the oxidant cumene hydroperoxide (CHP; 0.5 mM) and (E) the reductant dithiothreitol (DTT; 40 mM). (F) Dynamic range (DR) of the biosensor in U937 cells based on complete oxidation and reduction by CHP and DTT, respectively. (G) Ratiometric biosensor response over time for uninfected and M. tuberculosis-infected U937 Mφ. Error bars represent standard deviations from the means. *, *P* < 0.05; **, *P* < 0.01 (two-way ANOVA). Data are representative of results from at least three independent experiments performed in triplicate.

We exploited a noninvasive biosensor (Grx1-roGFP2) (roGFP, reduction-oxidation-sensitive green fluorescent protein) of GSH redox potential (*E_GSH_*) (24) to measure dynamic changes in the redox physiology of human macrophages (U937) upon infection with a virulent strain of M. tuberculosis (H37Rv). GSH is the most abundant low-molecular-weight thiol produced by mammalian cells; therefore, *E_GSH_* measurement provides a reliable and sensitive indicator of the cytoplasmic redox state of macrophages (20, 24). The biosensor shows an increase in the fluorescence excitation ratio at 405/488 nm upon oxidative stress, whereas a ratiometric decrease is associated with reductive stress (Fig. 1A). These ratiometric changes can be easily fitted into the modified Nernst equation to precisely calculate *E_GSH_* values (24).

U937 monocytes expressing cytosolic Grx1-roGFP2 (U937/Grx1-roGFP2) were differentiated to macrophages by the use of phorbol 12-myristate 13-acetate (PMA) and were infected with M. tuberculosis H37Rv (Fig. 1B). At various time points postinfection (p.i.), 405/488 ratios were measured by flow cytometry to calculate intracellular *E_GSH_* levels as described previously (20). We first confirmed the response of the biosensor to a well-known oxidant, cumene hydroperoxide (CHP), and a cell-permeable thiol reductant, dithiothreitol (DTT). As expected, the treatment of U937/Grx1-roGFP2 with CHP increased the 405/488 ratio, which corresponded to *E_GSH_* of −240 mV, and treatment with DTT decreased the 405/488 ratio, which corresponded to *E_GSH_* of −320 mV (Fig. 1C to F). Next, we examined the biosensor response upon infection with M. tuberculosis H37Rv. Uninfected U937/Grx1-roGFP2 cells exhibited a highly reduced cytoplasm level (405/488 ratio, ∼0.1 to 0.15 over time; *E_GSH_*, −301 ± 2 mV) (Fig. 1G). In contrast, M. tuberculosis infection gradually increased the biosensor oxidation ratio over time, which resulted in an approximately +20 mV shift in *E_GSH_* (−282 ± 2 mV) at 24 h p.i. (Fig. 1G).

**FIG 2:**
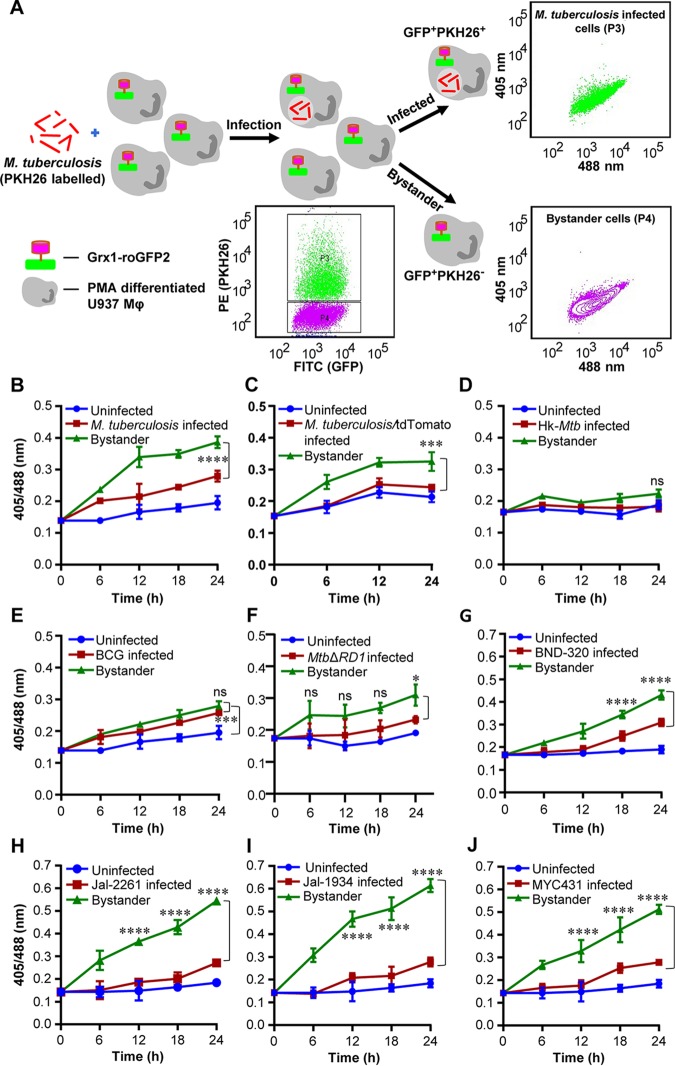
M. tuberculosis induces higher oxidative shift in *E_GSH_* of bystander Mφ than in that of infected U937 Mφ. (A) The M. tuberculosis H37Rv bacilli were stained with PKH26 membrane-staining dye and were used to infect PMA-differentiated U937/Grx1-roGFP2 Mφ at an MOI of 10. The sensor response was measured by flow cytometry. Based on the PKH26 fluorescence emitted by M. tuberculosis inside Mφ, the U937/Grx1-roGFP2 cells were gated into infected (P3) and bystander (P4) subpopulations. Dot plots show M. tuberculosis-infected (PKH26^-ve^/GFP^+ve^) and bystander (PKH26^-ve^/GFP^+ve^) U937/Grx1-roGFP2 Mφ at 24 h. FITC, fluorescein isothiocyanate. (B) The line graph shows biosensor responses at various time points in uninfected, M. tuberculosis-infected, and bystander U937/Grx1-roGFP2 cells. (C) M. tuberculosis stably expressing a red fluorescent protein, tdTomato (M. tuberculosis/tdTomato), was used to infect U937/Grx1-roGFP2 (MOI of 10), and the biosensor response of uninfected, infected, and bystander cells was measured over time. (D to J) Biosensor response of infected and bystander U937/Grx1-roGFP2 cells upon infection with PKH26-labeled (D) heat-killed M. tuberculosis (Hk-*Mtb*), (E) M. bovis BCG, (F) M. tuberculosis *ΔRD1*, (G) BND-320, (H) Jal-2261, (I) Jal-1934, and (J) MYC431. Error bars represent standard deviations from the means. ***, *P* < 0.001; ****, *P* < 0.0001 (two-way ANOVA). Data are representative of results from at least three independent experiments performed in triplicate.

In order to investigate the relative contributions of infected versus uninfected cells on *E_GSH_* levels of macrophage population, we assessed the biosensor response of M. tuberculosis-infected U937 and bystander macrophages. M. tuberculosis cells stained with a lipophilic dye, PKH26, were used to infect U937/Grx1-roGFP2 at a multiplicity of infection (MOI) of 10. About 42.75% ± 4.05 of U937/Grx1-roGFP2 cells were infected with PKH26-labeled M. tuberculosis (PKH26 positive/GFP positive [PKH26^+ve^/GFP^+ve^]) (Fig. 2A). Interestingly, bystander U937 cells (PKH26^-ve^/GFP^+ve^) showed a greater 405/488 ratio than infected cells (PKH26^+ve^/GFP^+ve^) at each time point tested (Fig. 2B). At 24 h p.i., the *E_GSH_* of bystander cells was −276 ± 2 mV compared to −286 ± 2 mV in the case of M. tuberculosis-infected cells (Fig. 2B). This suggests that the redox physiology of infected and bystander macrophages is distinctly affected during M. tuberculosis infection. Additionally, U937/Grx1-roGFP2 cells infected with M. tuberculosis genetically expressing a red fluorescent protein (RFP) (RFP:tdTomato) confirmed a higher 405/488 ratio of bystander cells (RFP negative/GFP positive [RFP^-ve^/GFP^+ve^]) than of infected cells (RFP^+ve^/GFP^+ve^) (Fig. 2C). Infection of U937/Grx1-roGFP2 with PKH26-labeled heat-killed M. tuberculosis (Hk-*Mtb*) did not increase oxidative stress in the infected or bystander cells (Fig. 2D), indicating that processes such as secretion of bioactive lipids or proteins are the likely modulators of intramacrophage *E_GSH_*. The secretory proteins of the ESX-1 family of M. tuberculosis are known to induce oxidative stress in infected macrophages (25). In agreement with this, the Mycobacterium bovis bacillus Calmette-Guérin (BCG) strain and the M. tuberculosis *ΔRD1* (*MtbΔRD1*) mutant, defective in secreting ESX-1 proteins (26), elicited a marginal degree of biosensor oxidation in infected or bystander U937/Grx1-roGFP2 cells (Fig. 2E and F). We measured the internalization of PKH26-labeled M. tuberculosis strains by flow cytometry and confirmed that in each case (M. tuberculosis, Hk-*Mtb*, and BCG), similar fractions (∼50%) of U937/Grx1-roGFP2 cells were infected. This precludes the influence of variations in the initial uptake rates on the *E_GSH_* of macrophages.

Since HIV-1-infected individuals are frequently coinfected with drug-resistant (DR) strains of M. tuberculosis (27, 28), we assessed the impact of single-drug-resistant (SDR) (BND-320), multidrug-resistant (MDR) (Jal-2261 and Jal-1934), and extensively drug-resistant (XDR) (MYC431) strains of M. tuberculosis isolated from patients (29) on *E_GSH_* of U937. Infection with BND-320 induced an increase in the fluorescence excitation 405/488 ratio of infected and bystander macrophages to a degree comparable to that seen with M. tuberculosis H37Rv (Fig. 2G). However, infection with MDR and XDR strains stimulated a significantly higher oxidative shift in *E_GSH_* of bystander cells than in infected U937 macrophages (Fig. 2H to J) . Also, oxidative stress was noticeably higher in bystander cells in the case of infection with MDR/XDR strains than with M. tuberculosis H37Rv (compare Fig. 2B with Fig. 2H to J). Altogether, these results confirm that infection with M. tuberculosis drives changes in *E_GSH_* of infected and bystander U937 cells and that clinical drug-resistant isolates are potent inducers of oxidative stress in macrophages.

### Reactivation of HIV-1 upon coculturing with M. tuberculosis-infected macrophages.

HIV-1 infects multiple immune cells, including macrophages, lymphocytes, and dendritic cells, whereas macrophages are the major host cells for M. tuberculosis. Therefore, how M. tuberculosis-infected macrophages communicate with HIV-1-infected cells of different origins remains unknown. The induction of oxidative stress in bystander cells raised the possibility that M. tuberculosis-infected macrophages might be able to reactivate virus by modulating redox physiology of neighboring cells that were chronically infected with HIV-1. This communication can be mediated either by direct cell-cell contact or, often, by bioactive soluble factors (e.g., cytokines). To assess these possibilities, we cocultured M. tuberculosis-infected U937 macrophages with J-Lat 10.6 lymphocytes, which harbor HIV-1 provirus in a latent state. Latent HIV-1 can be reactivated by various stimuli such as phorbol esters (12-O-tetradecanoylphorbol-13-acetate [TPA]), prostratin, and tumor necrosis factor alpha (TNF-α) (30). The integrated HIV-1 genome encodes GFP, which allows precise quantification of HIV-1 reactivation using flow cytometry. As expected, pretreatment of J-Lat with TNF-α (10 ng/ml) induced significant HIV-1 reactivation, which translated into the presence of greater percentage of GFP^+ve^ cells over time (see Fig. S1A in the supplemental material). Coculture of M. tuberculosis-infected U937 macrophages with J-Lat also showed a time-dependent increase in the level of GFP^+ve^ cells, indicating HIV-1 reactivation (Fig. S1A). However, coculture with either uninfected or Hk-*Mtb*-infected U937 macrophages significantly attenuated the induction of GFP from J-Lat cells (Fig. S1A). As an additional verification, we examined the effect of coculture on a monocytic cell line model (U1) of HIV-1 latency (31). The U1 cell line shows basal expression of two integrated copies of the HIV-1 genome, but gene expression and viral replication can be reactivated by various stimuli such as PMA, TNF-α, interferon gamma (IFN-γ), and granulocyte-macrophage colony-stimulating factor (GM-CSF) (32). We quantified HIV-1 reactivation by immunostaining for an HIV-1 core protein, p24, using flow cytometry. First, we confirmed that PMA treatment reactivated HIV-1 from U1 in a time-dependent manner (Fig. S1B). Second, similar to our findings in J-Lat, HIV-1 reactivation was readily observed upon coculture of U1 cells with M. tuberculosis-infected U937 (Fig. S1B). Coculture with uninfected U937 macrophages or Hk-*Mtb-*infected U937 macrophages, however, reactivated HIV-1 only partially (Fig. S1B).

10.1128/mBio.03293-19.1 FIG S1 M. tuberculosis-infected macrophages reactivate latent HIV-1 from lymphocytes and monocytes. Download FIG S1, PDF file, 0.6 MB. Copyright © 2020 Tyagi et al. 2020 Tyagi et al. This content is distributed under the terms of the Creative Commons Attribution 4.0 International license.

**FIG 3:**
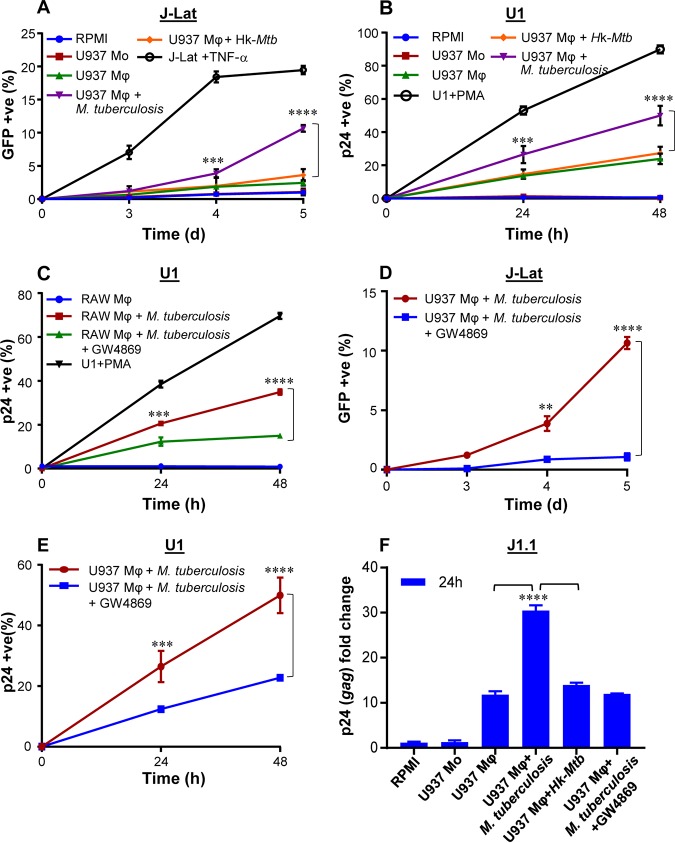
Culture supernatant derived from M. tuberculosis-infected macrophages (Mφ) reactivates HIV-1. (A and B) We determined the influence of culture supernatant derived from M. tuberculosis-infected Mφ on three latent cell lines of HIV-1: J-Lat, U1, and J1.1. U937 Mφ cells were infected with M. tuberculosis or Hk-*Mtb* (MOI of 10). At 24 h p.i., culture supernatant from the infected macrophages was collected, passed through a 0.2-μm-pore-size filter, and diluted in fresh RPMI medium (1:1 [vol/vol]). This supernatant was used to culture J-Lat and U1 cells, and HIV-1 reactivation was monitored over time by (A) measuring GFP fluorescence in J-Lat and (B) p24 immunostaining in U1 cells. As controls, we monitored HIV-1 reactivation from J-Lat and U1 cells cultured in the supernatant derived from U937 monocytes (Mo). HIV-1 reactivation upon treatment of J-Lat and U1 with TNF-α (10 ng/ml) and PMA (5 ng/ml), respectively, was taken as the positive control. d, days. (C) HIV-1 reactivation in U1 upon treatment with the supernatant derived from RAW264.7 Mφ cells infected with M. tuberculosis for 24 h. Treatment with the culture supernatant of uninfected RAW264.7 Mφ cells and treatment with PMA were used as the negative and positive controls for HIV-1 reactivation, respectively. (C to F) To investigate if supernatant-mediated HIV-1 reactivation is dependent on the presence of extracellular vesicles (e.g., exosomes), we treated M. tuberculosis-infected U937 and RAW264.7 Mφ cells with an inhibitor of exosome secretion (GW4869) for 24 h, followed by supernatant collection and reactivation of HIV-1 in (C [RAW] and E [U937]) U1, (D) J-Lat (U937), and (F) J1.1 (U937) cells as described in the text. HIV-1 reactivation was measured by p24 (*gag*) qRT-PCR in J1.1 cells. Error bars represent standard deviations from the means. **, *P* < 0.01; ***, *P* < 0.001; ****, *P* < 0.0001 (one-way ANOVA). Data are representative of results from at least two independent experiments performed in triplicate.

To examine if these findings may be related to the release of soluble factors from M. tuberculosis-infected macrophages, we treated J-Lat cells with culture supernatants derived from M. tuberculosis-infected U937 macrophages at 24 h p.i. A time-dependent increase in GFP expression was induced by the addition of supernatant (50% [vol/vol]) from M. tuberculosis*-*infected U937 to J-Lat cultures (Fig. 3A). Culture supernatant (50% [vol/vol]) from uninfected U937 or Hk-*Mtb*-infected macrophages showed diminished expression of GFP from J-Lat (Fig. 3A). Similarly to the results seen with J-Lat, treatment of U1 cells with the supernatant derived from M. tuberculosis-infected U937 macrophages reactivated HIV-1 to the highest level compared to supernatant from Hk-*Mtb*-infected or uninfected U937 (Fig. 3B). Marginal reactivation of HIV-1 by the supernatant of uninfected U937 macrophage was perhaps due to the known effect of PMA (used as a differentiating agent) on secretion of proinflammatory cytokines in the extracellular milieu (33). Consistent with this, supernatant from undifferentiated U937 monocytes completely failed to reactivate HIV-1 from U1 and J-Lat cells (Fig. 3A and B). To decisively rule out the influence of PMA on HIV-1 reactivation in our assays, we infected RAW264.7 murine macrophages with M. tuberculosis and collected supernatant at 24 h p.i. As a control, supernatant was also collected from uninfected RAW264.7 macrophages. Addition of the supernatant from uninfected RAW264.7 cells was completely ineffective in reactivation of HIV-1 from U1 cells, whereas supernatant derived from M. tuberculosis-infected RAW264.7 cells was potent in reactivating HIV-1 (Fig. 3C).

### Exosomes derived from M. tuberculosis-infected macrophages and mice reactivate HIV-1.

Reactivation of HIV-1 by the culture supernatant suggests a role for the extracellular vesicles (exosomes) frequently secreted by M. tuberculosis-infected macrophages. To investigate this possibility, we infected U937 and RAW264.7 macrophages with M. tuberculosis and treated infected macrophages with a well-known inhibitor of exosome secretion, GW4869 (34). At 24 h posttreatment with GW4869, we collected supernatant and performed HIV-1 reactivation in J-Lat and U1 cells, as described above. As shown in Fig. 3D and E, HIV-1 reactivation was observed in J-Lat and U1 in the case of treatment with supernatant derived from M. tuberculosis-infected macrophages, whereas a significant reduction in HIV-1 reactivation was detected in the case of addition of supernatant derived from GW4869-treated M. tuberculosis*-*infected macrophages. Furthermore, we employed a third cell line (J1.1 lymphocytes) latently infected with HIV-1 (35) and confirmed that only the supernatant derived from M. tuberculosis infected macrophages reactivated HIV-1 (as shown by *gag* reverse transcription-quantitative PCR [qRT-PCR]) compared to supernatant from uninfected, Hk-*Mtb*-infected and GW4869-treated macrophages (Fig. 3F). These results suggest a likely role for exosomes secreted by M. tuberculosis-infected macrophages in reactivating HIV-1. Since treatment with supernatant derived from M. tuberculosis-infected U937 and RAW264.7 macrophages resulted in identical degrees of HIV-1 reactivation, further experiments were conducted using RAW264.7 cells only. This was necessary to rule out any artefactual influence of PMA used to differentiate U937 monocytes prior to infection with M. tuberculosis. Furthermore, exosomes derived from M. tuberculosis-infected RAW264.7 have been shown to possess immune-modulatory properties comparable to those possessed by exosomes isolated from the serum of M. tuberculosis-infected mice or humans (36).

**FIG 4:**
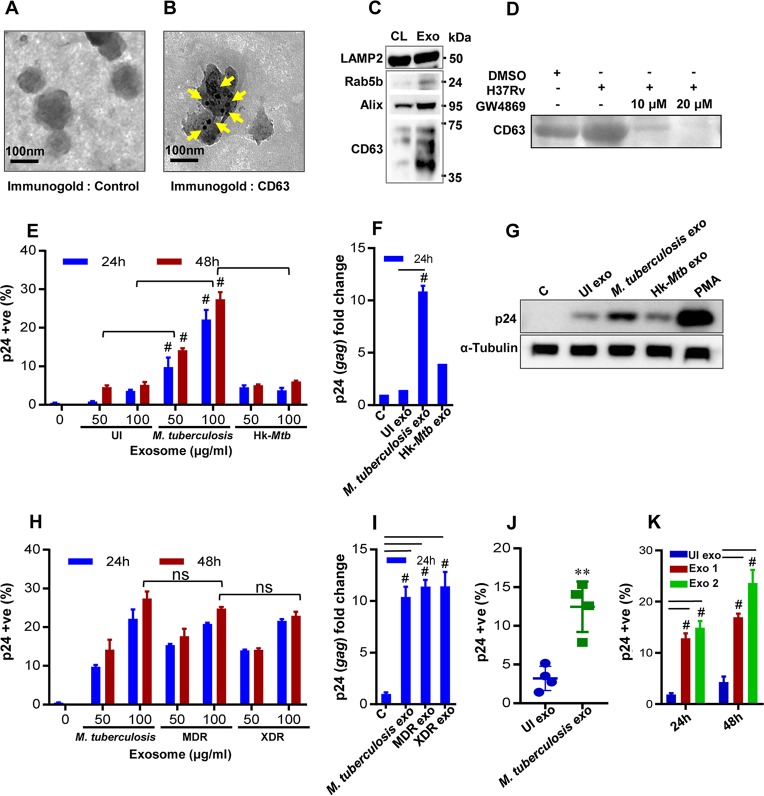
Exosomes derived from M. tuberculosis*-*infected macrophages (Mφ) and mice induce HIV reactivation. Exosomes were isolated from enriched cell culture supernatant of M. tuberculosis-infected RAW264.7 Mφ cells and were morphologically characterized by transmission electron microscopy (TEM). (A and B) Immunogold control (A) and CD63 (B) labeling of exosomes using the antibody against an exosome surface marker, CD63. Bars, 100 nm. (C) Immunoblot analysis of LAMP2, Rab5b, Alix, and CD63 in cell extract (CL; 30 μg) and purified exosomes (Exo; 30 μg) from M. tuberculosis*-*infected RAW264.7 Mφ. (D) Immunoblot analysis of exosome-specific marker CD63 to show dose-dependent reduction in exosome production by M. tuberculosis-infected RAW264.7 Mφ cells upon treatment with GW4869. Exosomes were derived from equal numbers of RAW264.7 Mφ in all cases, and equivalent volumes were loaded in all lanes. DMSO, dimethyl sulfoxide. (E) U1 cells were treated with 50 and 100 μg/ml of purified exosomes, and the level of HIV-1 reactivation was measured by flow cytometry using PE-labeled antibody specific to p24 (Gag) antigen at the indicated time points. (F) qRT-PCR for *gag* transcript at 24 h posttreatment with 100 μg/ml of purified exosomes. “C” and “UI exo” denote HIV-1 reactivation without any treatment or upon treatment with exosomes (100 μg/ml) derived from uninfected RAW264.7 Mφ, respectively. (G) Immunoblotting for p24 in the cell lysate (50 μg) at 24 h posttreatment with 100 μg/ml of purified exosomes. RAW264.7 Mφ cells were infected with MDR (Jal1934) and XDR (MYC431) strains for 24 h, and exosomes were isolated. (H and I) U1 cells were treated with exosomes, and HIV-1 reactivation was monitored at the indicated time by flow cytometry using (H) PE-labeled antibody specific to p24 (Gag) antigen and (I) qRT-PCR of *gag* transcript. (J and K) BALB/c mice were infected with 100 CFU of M. tuberculosis H37Rv or were left uninfected. Exosomes were isolated from mouse serum and from the lung tissue at 20 weeks p.i. using ExoQuick and quantified by micro-BCA assay. U1 cells were treated with exosomes derived from (J) serum (2 mg/ml) or (K) lungs (M. tuberculosis exo1 [100 μg/ml] and M. tuberculosis exo2 [200 μg/ml]), and HIV-1 reactivation was measured by flow cytometry using fluorescently tagged (PE-labeled) antibody specific to p24 (Gag) antigen. Error bars represent standard deviations from the means. Statistical analyses were performed using a two-tailed unpaired *t* test (J), one-way ANOVA (F and I), and two-way ANOVA (E, H, and K). ns, nonsignificant; **, *P* < 0.01; #, *P* < 0.001. Data are representative of results from at least two independent experiments performed in duplicate.

We infected RAW264.7 cells with M. tuberculosis H37Rv, and exosomes were isolated at 24 h p.i. using an ExoQuick Ultra system, which is reported to provide a high yield of pure exosomes (see Materials and Methods). Transmission electron microscopy (TEM) confirmed the diameter of the exosomes (*n* = 100) to be 80 to 150 nm (Fig. 4A; see Fig. S2A for wide-field images), which is consistent with previous studies (37). Immunogold labeling with CD63 primary antibody (Ab) followed by TEM confirmed the presence of this classical exosomes marker on the surface (Fig. 4B) (38). Immunoblot analysis performed to detect multiple exosome-specific markers such as Rab5b, Alix, and CD63 established their enrichment on the exosome fraction relative to cell lysate (Fig. 4C). LAMP2 was present in both fractions (Fig. 4C), as previously shown (38). Finally, we demonstrated that the pretreatment of M. tuberculosis-infected RAW264.7 macrophages with GW4869 significantly reduced the release of exosomes as revealed by the loss of the CD63 marker compared to untreated or M. tuberculosis-infected RAW264.7 macrophages (Fig. 4D). Altogether, we confirmed the isolation of exosomes from M. tuberculosis*-*infected macrophages to investigate redox-dependent activation of HIV-1.

10.1128/mBio.03293-19.2 FIG S2 Characterization of exosomes isolated from M. tuberculosis-infected macrophages and effect on HIV-1 reactivation and replication. Download FIG S2, PDF file, 0.1 MB. Copyright © 2020 Tyagi et al. 2020 Tyagi et al. This content is distributed under the terms of the Creative Commons Attribution 4.0 International license.

Various concentrations of exosomes isolated from M. tuberculosis-infected RAW264.7 macrophages were used to treat U1 cells, and HIV-1 reactivation was measured by p24 immunostaining and qRT-PCR analysis of *gag* transcript. Compared to the results seen with the uninfected control, M. tuberculosis-specific exosomes significantly induced HIV-1 reactivation from U1 cells. Results from immunostaining, immunoblotting, and qRT-PCR displayed an ∼4-fold increase in p24/*gag* levels compared to the uninfected control (Fig. 4E to G). Exosomes isolated from macrophages infected with Hk-*Mtb* or the *MtbΔRD1* mutant were significantly less effective in reactivating HIV-1 (Fig. 4E to G; see also Fig. S2B). The exosomes derived from RAW264.7 macrophages infected with MDR and XDR strains of M. tuberculosis were as potent as those derived from RAW264.7 macrophages infected with M. tuberculosis H37Rv in reactivating HIV-1 (Fig. 4H and I). To conclusively show that exosomes derived from M. tuberculosis infection reactivate HIV-1, we treated U1 with exosomes isolated from serum and lungs of mice chronically infected with M. tuberculosis and stained for p24. In each case, we observed significant (∼5-fold) reactivation of HIV-1 compared to exosomes isolated from uninfected animals (Fig. 4J and K). However, exosomes isolated from M. tuberculosis-infected lungs reactivated HIV-1 at a lower concentration than those isolated from serum (Fig. 4J and K).

Finally, it was previously reported that oxidative stress is associated with HIV-1 replication in primary human CD4^+^ T lymphocytes (39). Therefore, we isolated primary CD4^+^ T cells from human peripheral blood mononuclear cells (PBMCs), infected them with HIV-1 NL4-3 (T-cell-tropic virus), and measured HIV-1 replication upon addition of exosomes by the use of an HIV-1 p24 enzyme-linked immunosorbent assay (ELISA) at 5 days postinfection (Fig. S2C). The p24 ELISA confirmed that HIV-1-infected CD4^+^ T cells that had been treated with M. tuberculosis-specific exosomes showed a higher degree of virus replication than those that had been treated with Hk-*Mtb* or *MtbΔRD1* mutant exosomes (Fig. S2D). Taken together, our data suggest that exosomes could be among the important mediators of HIV-1 reactivation during M. tuberculosis infection.

### Exosomes from M. tuberculosis-infected macrophages modulate redox potential and gene expression in U1 cells.

**FIG 5:**
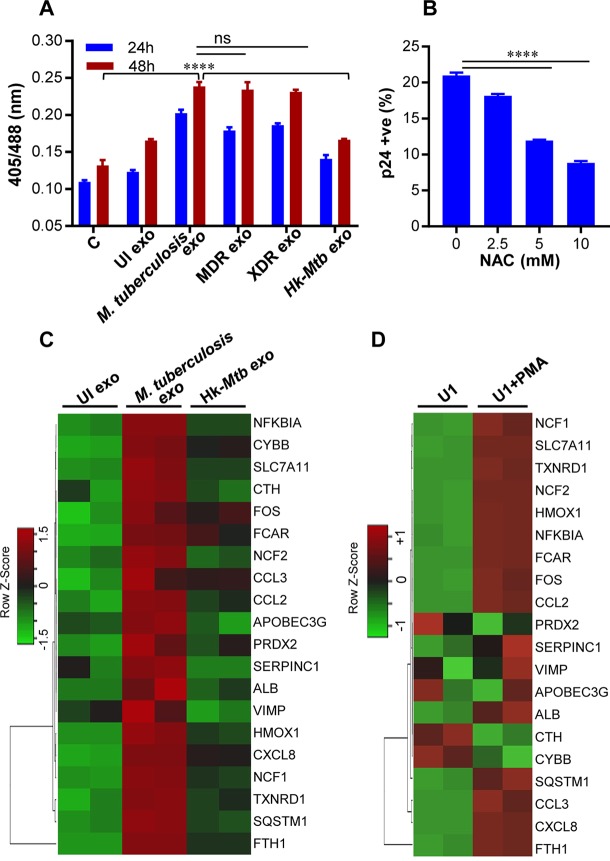
M. tuberculosis-specific exosomes reactivate HIV-1 by inducing oxidative stress. (A) RAW264.7 Mφ cells were infected with M. tuberculosis, Hk-*Mtb*, Jal1934 (MDR), and MYC431 (XDR) at an MOI of 10. At 24 h p.i., exosomes were isolated from the culture supernatant and exosomes (100 μg/ml) were used to treat U1/Grx1-roGFP2 for 24 and 48 h. Ratiometric biosensor response was measured by flow cytometry. “UI exo” denotes exosomes isolated from uninfected RAW264.7. (B) U1 cells were pretreated with N-acetyl cysteine (NAC) for 1 h at the indicated concentrations followed by exposure to M. tuberculosis-specific exosomes (100 μg/ml) for 24 h. HIV-1 reactivation was measured by flow cytometry using fluorescently tagged (PE-labeled) antibody specific to p24 (Gag) antigen. Data are representative of results from at least three independent experiments performed in triplicate. (C) U1 cells were treated with exosomes (100 μg/ml) isolated from uninfected, M. tuberculosis*-*infected, and Hk-*Mtb-*infected RAW264.7. Total RNA from U1 cells was isolated after 12 h of exosome treatment, followed by expression analysis of 185 genes specific to HIV host response and oxidative stress response utilizing NanoString customized gene expression panels. Data were normalized by nSolver (NanoString) software. Genes with significant changes were selected based on *P* values of <0.05 and fold change values of >1.5 for comparisons between M. tuberculosis and UI, M. tuberculosis and Hk-*Mtb*, and Hk-*Mtb* and UI. Data are displayed as heat maps of three groups analyzed together. (D) Expression profile of genes affected by M. tuberculosis-specific exosomes in U1 monocytes and PMA-treated U1 macrophages. Error bars represent standard deviations from the means. ****, *P* < 0.0001 (two-way ANOVA). Data are representative of results from at least two independent experiments performed in duplicate.

Elevated levels of ROS and reactive nitrogen intermediates (RNI) and depletion/oxidation of intracellular thiols (cysteine, thioredoxin, GSH) were shown to activate the HIV-1 long terminal repeat (LTR) through the activity of redox-responsive transcription factor NF-κB (40, 41). Therefore, we first tested whether exosomes derived from RAW 264.7 cells infected with various M. tuberculosis strains induced oxidative stress to reactivate HIV-1. Treatment of U1/Grx1-roGFP2 with exosomes derived from RAW 264.7 infected with M. tuberculosis H37Rv, MDR, and XDR strains uniformly increased biosensor ratios at 24 h and 48 h posttreatment, indicating oxidative stress (Fig. 5A). As expected, exosomes derived from uninfected or Hk-*Mtb*-infected RAW 264.7 cells failed to induce oxidative stress in U1 cells (Fig. 5A). To show that exosome-triggered oxidative stress precedes HIV-1 reactivation, we pretreated U1 cells with the GSH-specific antioxidant NAC, followed by addition of exosomes. Treatment with NAC abrogated HIV-1 reactivation by M. tuberculosis exosomes (Fig. 5B). This result reconfirms that oxidative stress is likely to be an important mechanism induced by M. tuberculosis-specific exosomes in reactivation of HIV-1.

Next, we examined the influence of M. tuberculosis-specific exosomes on host gene expression by the use of NanoString nCounter technology. This method permits the absolute quantification of a large number of RNA transcripts without any requirements for reverse transcription or DNA amplification (42). From the NanoString panel, we focused on 185 host genes that are known to respond to HIV-1 replication (e.g., HIV-1 receptors-ligands, proteins involved in HIV replication, inflammatory response, apoptosis, cell cycle, and transcription regulators of HIV-1 LTR) as well as oxidative stress-responsive genes (see Table S1a in the supplemental material). Exosomes derived from uninfected or M. tuberculosis (live or Hk)-infected RAW 264.7 macrophages were used to treat U1 cells, and total RNA was isolated at 12 h posttreatment. An early time point was chosen to ensure that we would capture gene expression changes that precede HIV-1 reactivation upon challenge with exosomes. Also, at a later time point, the primary effect of exosomes can be masked due to transcriptional changes in response to HIV-1 proliferation and associated cytopathic consequences. Since PMA is known to stimulate transcription of HIV-1 LTRs along with inflammation-related genes, we isolated RNA from U1 upon treatment with 5 ng/ml of PMA for 12 h as a control.

10.1128/mBio.03293-19.6 TABLE S1 NanoString nCounter gene expression data, list of genes, normalized intensities of differentially expressed genes, and fold changes of expression levels of differentially expressed genes. Download Table S1, XLSX file, 0.03 MB. Copyright © 2020 Tyagi et al. 2020 Tyagi et al. This content is distributed under the terms of the Creative Commons Attribution 4.0 International license.

As expected, PMA modulated the expression (fold change of >1.5; *P* < 0.05) of 72 genes related to oxidative stress, inflammation, and HIV-1 infection (Table S1). In contrast, M. tuberculosis-specific exosomes induced the expression of 19 genes compared to uninfected or Hk-*Mtb* exosomes (Table S1), indicating a specific rather than broad influence on host gene expression. Consistent with our biosensor data, treatment with M. tuberculosis exosomes induced expression of genes encoding components of superoxide-producing enzyme, i.e., NADPH oxidase (e.g., cytochrome B-245 beta chain [CYBB], NCF1, and NCF2) (Fig. 5C) compared to treatment with Hk-*Mtb* or uninfected exosomes. As a consequence, several genes involved in mitigating the effects of ROS and maintaining redox balance, such as PRDX2 (peroxiredoxin) (43), TXNRD1 (thioredoxin reductase 1) (44), sodium-dependent cysteine-glutamate antiporter (SLC7A11) (45), and ferritin (FTH1) (46), were induced specifically in U1 cells treated with M. tuberculosis exosomes (Fig. 5C). Additionally, a gene encoding heme oxygenase 1 (HMOX1) was induced in U1 treated with M. tuberculosis exosomes (Fig. 5C). HMOX1 is involved in heme catabolism, and its dysregulation and polymorphism in its promoter are linked to HIV-associated neurocognitive disorder (47). A gene encoding a member of the selenoprotein family (VCP-interacting membrane protein [VIMP]; also known as selenoprotein S) was induced by M. tuberculosis exosomes (Fig. 5C). VIMP is a redox-sensing protein that regulates inflammation by mediating cytokine production (48), suggesting that VIMP can coordinate HIV-1 reactivation by triggering an inflammatory reaction in response to oxidative stress induced by M. tuberculosis exosomes.

Since M. tuberculosis exosomes reactivated HIV-1, genes affecting transcription of HIV-1 LTR were induced. For example, NFKBIA and SQSTM1/P62, involved in regulating transcription factor NF-κB, were upregulated (49) (Fig. 5C). Another transcription factor, FOS, which reactivates HIV-1 (50), was also induced by M. tuberculosis exosomes. Several genes encoding host inflammatory mediators were induced by M. tuberculosis exosomes. For example, expression of CXC chemokine subfamily-8 (CXCL8), monocyte chemoattractant protein 1 (MCP1; CCL2) and macrophage inflammatory protein-1 alpha (MIP-1a; CCL3) showed higher levels of expression in U1 treated with M. tuberculosis-specific exosomes than were seen with Hk-*Mtb*-infected or uninfected exosomes (Fig. 5C). All of these factors are well known to facilitate HIV-1 infection and promote replication in macrophages (51). In fact, higher levels of CCL2 were detected in the bronchoalveolar lavage (BAL) fluid of pulmonary TB patients and pleural fluid of HIV-1-infected patients, indicating the importance of this proinflammatory cytokine in HIV-M. tuberculosis coinfection (52). Interestingly, an apolipoprotein B mRNA editing enzyme, catalytic polypeptide-like 3G (APOBEC3G), a known HIV restriction factor (53), was also induced by M. tuberculosis exosomes. In addition to viral restriction, APOBEC3G activity also promotes heterogeneity in HIV sequence, resulting in HIV phenotypes with a greater capacity to escape immune pressures (54). Therefore, it is likely that coinfection with M. tuberculosis and subsequent release of exosomes can accelerate APOBEC3G-mediated generation of fitter viral variants. Consistent with this, pleural fluid mononuclear cells (PFMCs) from HIV-M. tuberculosis-coinfected patients show higher levels of expression of APOBEC3G (55). Finally, a gene encoding serpin peptidase inhibitor, clade C (SERPINC1; antithrombin), which is induced at very early stages of HIV-1 replication and is a component of the viral core (56), was upregulated by M. tuberculosis exosomes (Fig. 5C). We found a noticeable similarity between the transcriptional signatures induced by M. tuberculosis exosomes and by PMA treatment (Fig. 5D). This is in agreement with an earlier report showing increased oxidative stress in U1 cells upon PMA treatment (20). Taken together, our results show that exosomes released by M. tuberculosis-infected macrophages modulate host redox metabolism and inflammatory responses to ensure HIV-1 reactivation.

### Influence of M. tuberculosis-specific exosomes on oxidative phosphorylation (OXPHOS) of U1 cells.

**FIG 6:**
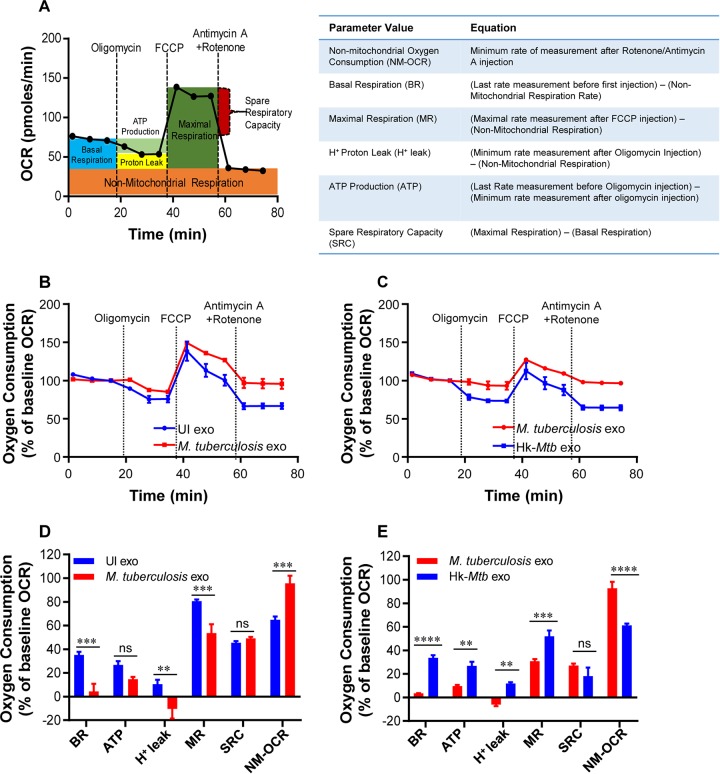
M. tuberculosis-specific exosomes modulate OXPHOS of U1 cells. (A) Schematic presentation of Agilent Seahorse XF Cell Mito Stress test profile of the key parameters of mitochondrial respiration. (B and C) RAW264.7 Mφ were infected with M. tuberculosis (B) and Hk-*Mtb* (C) at an MOI of 10. At 24 h p.i., exosomes were isolated from the culture supernatant, and exosomes (100 μg/ml) were used to treat U1 for 48 h. Respiratory profiles of U1 cells upon treatment with exosomes isolated under the indicated conditions are shown. UI exo, exosomes isolated from uninfected RAW264.7. Respiratory profiles were measured, and data are expressed as percent basal oxygen consumption rate (% of baseline OCR). Oxygen consumption was measured without any inhibitor (basal respiration), followed by measurement of OCR change upon sequential addition of oligomycin (ATP synthase inhibitor) (1 μM) and cyanide-4 (trifluoromethoxy]phenylhydrazone [FCCP) (0.25 μM), which uncouples mitochondrial respiration and maximizes OCR. Finally, respiration was completely shut down by inhibiting respiration using antimycin A and rotenone (0.5 μM each), which inhibit complexes III and I, respectively. (D and E) Various respiratory parameters as outlined in the table in panel A were measured for M. tuberculosis (D) and Hk-*Mtb* (E) using the values obtained from the data set depicted in panels B and C and as described in Materials and Methods. Error bars represent standard deviations from the means. ns, nonsignificant; **, *P* < 0.01; ***, *P* < 0.001; ****, *P* < 0.0001 (two-tailed unpaired *t* test). Data are representative of results from at least two independent experiments performed in triplicate.

We have previously shown that HIV-1 reactivation precedes an oxidative shift in mitochondrial *E_GSH_* levels (20). This indicates that M. tuberculosis exosomes might influence the mitochondrial physiology of HIV-infected cells to promote oxidative stress and viral replication. To examine this possibility, we measured several key parameters associated with oxidative phosphorylation (OXPHOS) using Seahorse XF Flux technology (Fig. 6A).

Several parameters of mitochondrial respiration, including basal respiration (Basal-Resp), ATP-linked respiration, proton leakage, and spare respiratory capacity (SRC), were derived by the successive addition of pharmacological agents to exosome-challenged U1 cells, as outlined in Fig. 6A. To determine each parameter, determinations of three reiterated oxygen consumption rates (OCR) were performed over an 80-min period. First, the baseline cellular OCR was measured, after which Basal-Resp was derived by subtracting nonmitochondrial respiration. Second, an inhibitor of complex V (oligomycin) was added, and the resulting OCR is used to calculate the ATP-linked OCR (by deducting the OCR from the baseline cellular OCR after oligomycin addition) and the proton leakage rate (by subtracting nonmitochondrial respiration from the OCR upon oligomycin addition). Third, the maximal respiration (Max Resp) rate, which represents the change in the OCR after uncoupling ATP synthesis from electron transport (ET) by adding carbonyl cyanide-4-(trifluoromethoxy)phenylhydrazone (FCCP), was determined. Finally, antimycin A, a complex III inhibitor, and rotenone, a complex I inhibitor, were added together to inhibit electron transport chain (ETC) function, revealing the nonmitochondrial respiration (non-Mito Resp) rate. SRC was calculated by subtracting basal respiration from the maximal respiratory capacity.

U1 cells were treated with exosomes isolated from RAW264.7 cells infected with viable *M. tuberculosis* or Hk-*Mtb* for 48 h. Following this, U1 cells were seeded onto the XF cartridge plates and subjected to the mitochondrial stress test to measure various OXPHOS parameters as described above. Compared to the results seen with exosomes from uninfected macrophages, treatment of U1 cells with M. tuberculosis-specific exosomes significantly decreased the levels of various respiratory parameters, including basal respiration, ATP-linked OCR, and H^+^ leakage (Fig. 6B and D). In contrast, the non-Mito Resp rate was significantly increased, whereas the SRC was not significantly affected (Fig. 6B and D). Exosomes derived from macrophages infected with Hk-*Mtb* modulated OXPHOS parameters comparably to exosomes from uninfected macrophages (Fig. 6C and E). The contrasting influences of viable *M. tuberculosis* and Hk-*Mtb* exosomes on Basal-Resp, ATP-linked OCR, and proton leakage indicated a profound deceleration of respiration of U1 upon reactivation of HIV-1 by M. tuberculosis exosomes. Generation of ROS (e.g., superoxide) is an inevitable consequence of normal mitochondrial respiration. Since M. tuberculosis exosomes induce an oxidative shift in *E_GSH_* of U1, deceleration in Basal-Resp and ATP-linked OCR could represent cellular strategies invoked to avoid overwhelming oxidative stress. This would ensure successful HIV-1 reactivation without triggering the detrimental effects of ROS on U1 cells. In line with our findings, active HIV-1 replication depends largely on increased glycolytic flux rather than OXPHOS to meet the surge in the demand for biosynthesis and bioenergetics (57).

Our OXPHOS results also provide clues about the mechanism of oxidative shift in *E_GSH_* upon HIV reactivation by exosomes. We observed a significant increase in nonmitochondrial O_2_ consumption in the case of U1 cells treated with M. tuberculosis*-*specific exosomes (Fig. 6D and E). Nonmitochondrial O_2_ consumption is usually due to activities of enzymes associated with inflammation such as lipoxygenase, cyclo-oxygenase, and NADPH oxidase (58). Consistent with this, our NanoString data showed increased expression of genes encoding various components of the NADPH oxidase complex. Altogether, HIV-1 reactivation by M. tuberculosis-specific exosomes was associated with a marked change in OXPHOS parameters, including reductions in basal OCR and ATP-linked OCR. An increase in the nonmitochondrial OCR is likely responsible for the generation of oxidative stress during HIV-M. tuberculosis coinfection.

### Proteomics of exosomes released upon M. tuberculosis infection.

Having established that M. tuberculosis-specific exosomes reactivate HIV-1 by modulating redox and bioenergetics, we sought to determine the content of exosomes. Mycobacterial components (e.g., lipids, proteins, and RNA) are consistently enriched in the exosomes of M. tuberculosis-infected macrophages (59, 60). However, the host proteins within exosomes isolated from M. tuberculosis-infected macrophages remain uncharacterized. This is important as immune-activated macrophages secrete several redox-signaling proteins involved in eliciting a proinflammatory response and oxidative stress in the neighboring cells (43). On this basis, we reasoned that profiling of exosome-associated host proteins will likely provide new insight into how M. tuberculosis induces HIV reactivation.

**FIG 7:**
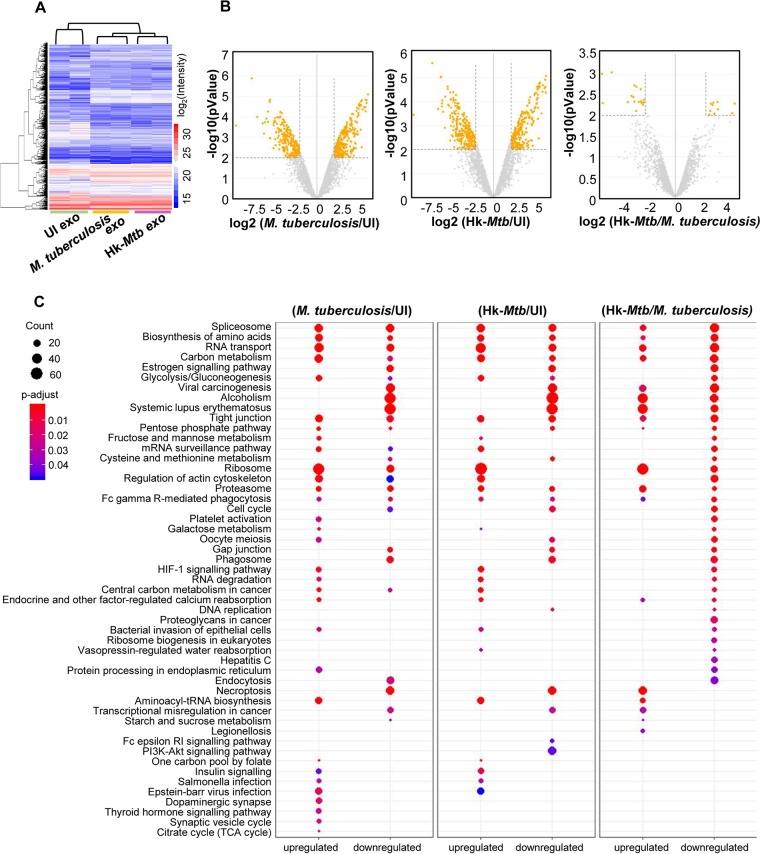
Proteomics of M. tuberculosis-specific exosomes by LC-MS/MS. RAW264.7 Mφ cells were infected with M. tuberculosis and Hk-*Mtb* at an MOI of 10 or left uninfected (UI). At 24 h p.i., exosomes were isolated from the culture supernatant for LC-MS/MS. (A) Heat map of differentially expressed proteins of exosomes in three samples. (B) Volcano plots of differentially expressed proteins. Significantly upregulated and downregulated proteins with log_2_ fold change values of more than 2 are shown as orange dots. (C) Enriched KEGG signaling pathways with proteins upregulated or downregulated in different comparison groups as indicated. PI3K, phosphatidylinositol 3-kinase.

We identified proteins associated with exosomes from uninfected, live M. tuberculosis-infected, and Hk-*Mtb*-infected RAW264.7 cells by the use of liquid chromatography-tandem mass spectrometry (LC-MS/MS) (Fig. 7A). Consistently, we observed a lower quantity of exosomes from uninfected and Hk-*Mtb*-infected samples than from live M. tuberculosis-infected samples. Therefore, we matched the amount of exosomes (30 μg) in all three samples by increasing the concentration of exosomes derived from uninfected and Hk-*Mtb*-infected macrophages. In total, 4,953 proteins were identified in all three samples with two biological replicates (Sequest Program) (Fig. 7A; see also Table S2). As expected, a high level of correlation (>0.90, Pearson correlation coefficient) was observed between two biological replicates from the same group than between two biological replicates from unrelated groups (0.6 to 0.7) (Fig. S3). About 80 of the 100 most extensively identified exosomal proteins listed in the ExoCarta database were identified in our data set. While ∼3,250 proteins overlapped among the three groups, we discovered that 86, 298, and 142 proteins were exclusively present in the uninfected, live M. tuberculosis-infected, and Hk-*Mtb*-infected groups, respectively (Fig. S3). Analysis of differentially expressed proteins (Table S3a to c) (log_2_ fold; *P* ≤ 0.01) showed that 436 proteins were upregulated and 290 were downregulated in live M. tuberculosis-infected exosome samples in comparison to uninfected samples (Fig. 7B). Similarly, 390 proteins were induced and 337 were repressed in Hk-*Mtb*-infected samples compared to uninfected samples (Fig. 7B). Direct comparison of live M. tuberculosis versus Hk-*Mtb* infection results revealed that only ∼40 proteins were differentially enriched in exosomes derived from live M. tuberculosis-infected macrophages (Fig. 7B). The fact that infection with Hk-*Mtb* resulted in decreased exosome release indicates that major differences lie in the quantity rather than the quality of host proteins in exosomes derived from live M. tuberculosis-infected versus Hk-*Mtb*-infected samples.

10.1128/mBio.03293-19.3 FIG S3 Proteomics of M. tuberculosis-specific exosomes determined by LC-MS/MS. Download FIG S3, PDF file, 0.1 MB. Copyright © 2020 Tyagi et al. 2020 Tyagi et al. This content is distributed under the terms of the Creative Commons Attribution 4.0 International license.

10.1128/mBio.03293-19.7 TABLE S2 List of proteins identified in LC/MS-MS analysis of uninfected, live H37Rv (M. tuberculosis) and heat-killed H37Rv (Hk-*Mtb*) exosomes. Download Table S2, XLSX file, 5.0 MB. Copyright © 2020 Tyagi et al. 2020 Tyagi et al. This content is distributed under the terms of the Creative Commons Attribution 4.0 International license.

10.1128/mBio.03293-19.8 TABLE S3 List of differentially expressed proteins, biological process gene ontology (GO) term enrichment analysis, cell localization gene ontology (GO) term enrichment analysis, and molecular function gene ontology. Download Table S3, XLSX file, 0.4 MB. Copyright © 2020 Tyagi et al. 2020 Tyagi et al. This content is distributed under the terms of the Creative Commons Attribution 4.0 International license.

Biological pathway analysis by Gene Ontology (GO) showed that the differentially expressed proteins belonged to diverse categories, including cellular/metabolic processes, biological regulation, and response to stimulus (Fig. S4). Molecular function analysis by GO revealed that most of the proteins carry out binding and catalytic activity, indicating their roles in exosome cargo sorting and in release and uptake by the recipient cells (Fig. S5). This was further confirmed by cell component analysis wherein exosomal proteins were found to belong mainly to cell organelle, membrane, and macromolecular complexes involved in exosomes biogenesis (Fig. S5; see also Table S3).

10.1128/mBio.03293-19.4 FIG S4 Functional classification of differentially expressed proteins of exosomes. Download FIG S4, PDF file, 2.5 MB. Copyright © 2020 Tyagi et al. 2020 Tyagi et al. This content is distributed under the terms of the Creative Commons Attribution 4.0 International license.

10.1128/mBio.03293-19.5 FIG S5 Molecular function and cellular component analysis of differentially expressed proteins in exosomes. Download FIG S5, PDF file, 2.2 MB. Copyright © 2020 Tyagi et al. 2020 Tyagi et al. This content is distributed under the terms of the Creative Commons Attribution 4.0 International license.

Next, we classified the differentially expressed proteins of each group by KEGG signaling pathway (Table S4) by use of the KEGG database (Fig. 7C). M. tuberculosis exosomes were found to be enriched with proteins involved in glycolysis, gluconeogenesis, fructose and mannose metabolism, galactose metabolism, pentose phosphate pathway (PPP), and cysteine and methionine metabolism. Increased oxidative stress in HIV-infected patients is associated with higher glucose utilization and deficiency of cysteine and methionine (61–63). Enrichment of sugar metabolic enzymes in M. tuberculosis exosomes possibly assists in HIV-1 reactivation by fueling ATP generation processes for energy-consuming functions such as virus transcription, translation, packaging, and release. Similarly, cysteine metabolism serves as a source of GSH biogenesis, while PPP enzymes provide NADPH for regenerating GSH from oxidized GSH (GSSG). Both of these activities are essential for alleviating excessive oxidative stress to avoid cell death during HIV-1 reactivation (16). We observed an enrichment of proteins coordinating RNA transport/quality control, DNA replication, and the cell cycle, all of which are important for the reactivation of HIV-1 (64, 65). Importantly, levels of estrogen signaling proteins involved in induction of mitochondrial ROS and HIV-1 reactivation are enriched in M. tuberculosis exosomes (66, 67). HIF-1 signaling plays an important role in HIV-1 pathogenesis by facilitating viral replication and promoting lymphocyte and macrophage-mediated inflammatory responses (68). HIF-1 pathway proteins are specifically induced in M. tuberculosis exosomes (Fig. 7C).

10.1128/mBio.03293-19.9 TABLE S4 KEGG pathway enrichment analysis of proteins in heat killed H37Rv (H) exosomes versus live H37Rv (R) exosomes, heat-killed H37Rv (H) exosomes versus uninfected (U) exosomes, and live H37Rv (R) exosomes versus uninfected (U) exosomes. Download Table S4, XLSX file, 0.03 MB. Copyright © 2020 Tyagi et al. 2020 Tyagi et al. This content is distributed under the terms of the Creative Commons Attribution 4.0 International license.

**FIG 8:**
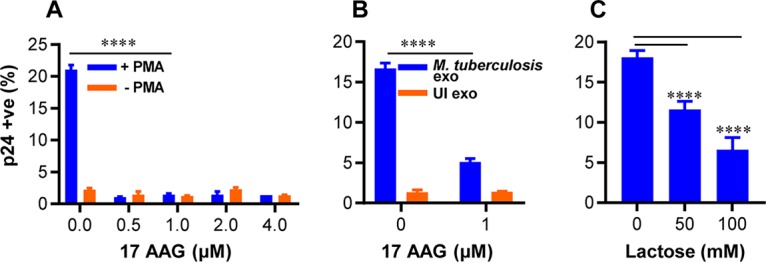
Hsp-90 and galectin inhibitors reverse exosome-mediated HIV-1 reactivation in U1 cells. (A) U1 cells were stimulated with 5 ng/ml of PMA to induce HIV-1 reactivation in the presence of the indicated concentrations of 17-AAG (Hsp-90 inhibitor). (B) U1 cells were treated with 100 μg/ml of exosomes isolated from uninfected (UI) or M. tuberculosis-infected RAW264.7 Mφ as described in the text, and HIV-1 reactivation was monitored in the presence of the indicated concentration of 17-AAG. (C) M. tuberculosis-infected RAW264.7 macrophages were treated with 50 and 100 mM lactose (galectins inhibitor) for 24 h, and exosomes were isolated. Those exosomes were then used to reactivate HIV-1 from U1 cells. HIV-1 reactivation was measured by flow cytometry using fluorescently tagged (PE-labeled) antibody specific to p24 (Gag) antigen. Error bars represent standard deviations from the means. ****, *P* < 0.0001 (two-way ANOVA [A and B] and one-way ANOVA [C]). Data are representative of results from at least two independent experiments performed in duplicate.

We discovered that members of the heat shock protein (HSP) family and galectins were enriched in both live M. tuberculosis and Hk-*Mtb* exosomes. However, M. tuberculosis exosomes showed the exclusive presence of Cdc37, which functions as a cochaperone for Hsp90 (69). Hsp90 promotes HIV-1 reactivation from latently infected cell lines and CD4^+^ T cells (70). Likewise, galectins are consistently associated with oxidative stress, inflammation, and HIV-1 reactivation (71, 72). Also, the level of STAT-1 of the JAK-STAT signaling pathway, which is well known to modulate HIV replication cycles, was enriched in M. tuberculosis-specific exosomes (73, 74). Finally, to assess the biological relevance of these findings, we examined the importance of HSPs and galectins in HIV-1 reactivation by M. tuberculosis exosomes. We used a specific inhibitor of Hsp90, namely, 17-(N-allylamino)-17-demethoxygeldanamycin (17-AAG) (70), for this purpose. Hsp90 is well known to modulate exosome release (75). Thus, rather than pretreating M. tuberculosis-infected RAW264.7 with 17-AAG, which is likely to affect exosome secretion, we studied the influence of 17-AAG on exosome-mediated HIV-1 reactivation in U1. We found that treatment of U1 with 17-AAG uniformly subverted the reactivation of HIV-1 by PMA or M. tuberculosis-specific exosomes (Fig. 8A and B). Galectins form an inhibitory complex with lactose via their carbohydrate binding domain (76). We treated M. tuberculosis-infected RAW 264.7 macrophages with 50 and 100 mM lactose for 24 h to inhibit expression of cellular galectins followed by exosome isolation. As indicated in Fig. 8C, exosomes isolated from the lactose-treated cells showed significantly reduced HIV-1 reactivation. We envision that the proteomics data generated in this study will facilitate future experimentation designed to understand the contribution of host factors in HIV-1 reactivation during HIV-M. tuberculosis coinfection. Altogether, infection with viable M. tuberculosis induces secretion of specific proteins in exosomes to reactivate HIV-1 by affecting redox, central metabolism, and inflammatory responses.

## DISCUSSION

We have previously shown that a marginal oxidative shift in *E_GSH_* is sufficient to reactivate HIV-1 (20). Others have shown that M. tuberculosis induces oxidative stress and GSH imbalance in infected macrophages, animals, and humans (21, 22). HIV-M. tuberculosis-coinfected patients suffer from glutathione stress, metabolic deficiencies, and immune dysfunction (21). In this study, we made an effort to unify into a coherent picture these separate observations, establishing a functional link between M. tuberculosis-induced oxidative stress and HIV-1 reactivation from latency. Furthermore, we provide evidence for oxidative stress-mediated HIV-1 reactivation, which relies on the secretion of biological effectors present in the exosomes released from M. tuberculosis*-*infected macrophages. Although the connections between M. tuberculosis, oxidative stress, and HIV-1 reactivation might appear obvious in hindsight, intracellular redox metabolism is dependent on many pathways. HIV-1 reactivation is a multifactorial process; hence, we expect multiple mechanisms to be in play with links to redox metabolism. In fact, our NanoString data suggest that the effect of M. tuberculosis-specific exosomes on HIV reactivation is likely mediated by diverse genetic factors. For example, superoxide generation by NADPH oxidase could be one of the factors contributing to an oxidative shift in *E_GSH_* and HIV-1 reactivation upon treatment with exosomes. However, the induction of several genes encoding antioxidant enzymes contradicts the requirement of ROS for viral reactivation. It appears that cells attempt to mitigate excess ROS to ensure exosome-mediated HIV-1 reactivation without triggering global ROS-mediated cytotoxicity. Consistent with this, treatment with M. tuberculosis-specific exosomes induces only a modest (−276 mV) oxidative shift in *E_GSH_* of U1 cells. Similar oxidative changes in *E_GSH_* were earlier found to uniformly reactivate HIV-1 from latency without affecting cellular viability (20). Low levels of ROS are known to activate HIV LTR via activation of NF-κB. In agreement with this, expression data confirmed the induction of NF-κB-IA and SQSTM1/P62 involved in regulating NF-κB activation upon exosome treatment. The induction of AP-1 (FOS), which is activated by ROS and reactivates HIV-1 (50), also indicates that exosome-promoted oxidative stress acts as a critical cue to reactivate HIV-1 via redox-sensitive transcription factors. This explanation aligns well with the ability of the antioxidant NAC to subvert HIV-1 reactivation by exosomes.

Despite the importance of ROS in HIV-1 reactivation, we lack an understanding of the source of intracellular ROS in HIV-infected cells. Since the respiratory chain is the major site for generation of ROS such as superoxide, cell flux assays showing M. tuberculosis exosome-mediated deceleration of mitochondrial OCR provide new mechanistic insight. It is well known that reduced mitochondrial OCR leads to build-up of NADH, which results in the trapping of flavin mononucleotide (FMN) in the reduced state on complex I of the ETC (77). Reduced FMN has been consistently shown to donate one electron to O_2_, resulting in the generation of ROS (i.e., superoxide) by complex I (77). Additionally, we discovered that nonmitochondrial OCR is significantly induced by M. tuberculosis-specific exosomes, which links oxidative stress to activities of enzymes unrelated to mitochondria (e.g., NADPH oxidase, lipoxygenase, and cyclo-oxygenase) as ROS sources (58). All of these enzymes are influenced by HIV infection and are known to generate ROS and influence GSH homeostasis (78–80). Importantly, a recent study showed that treatment of bone marrow-derived macrophages (BMDM) with exosomes derived from M. tuberculosis*-*infected macrophages increases recruitment of NADH oxidase on the phagosomes (60). Taken together, the data suggest that both mitochondrial and nonmitochondrial mechanisms associated with oxygen consumption likely mediate ROS generation and HIV-1 reactivation by M. tuberculosis exosomes. Our proteomics data identified several host proteins associated with HIV-1 reactivation in M. tuberculosis exosomes (e.g., galectins, HSPs, Cdc37). Hsp90 is abundantly present in the serum of HIV-M. tuberculosis-coinfected patients (81). Galectins such as Gal3 secrete into exosomes (82) and promote redox imbalance (83), whereas Gal9 is frequently found in the plasma of TB patients (84) and reactivates HIV-1 (71). While we have characterized the proteome of M. tuberculosis exosomes derived from macrophages, it is certain that the presence of immunomodulatory proteins, lipids, and RNA of M. tuberculosis also influences HIV-1 reactivation. In this context, findings obtained using the *MtbΔRD1* mutant suggest that proteins secreted by the ESX-1 system are important for redox-dependent reactivation of HIV-1. Several mycobacterial proteins (e.g., Ag85, Mpt32/64, and HspX), including those secreted via the ESX-1 system (e.g., Esat-6 and Cfp-10), were detected in exosomes during M. tuberculosis infection (85). Furthermore, host ESCRT machinery (ESCRT III) that participates in exosome formation is recruited onto M. tuberculosis-containing phagosomes in an ESX-1-dependent manner (86). Likewise, ESX-1 modulates the presence of Hsp90 and Gal3 on M. tuberculosis phagosomes (86, 87), suggesting the importance of the ESX-1 secretion system in exosome biogenesis and in the abundance/localization of Hsp90 and Gal3.

It can be argued that the exosomes released by M. tuberculosis*-*infected alveolar macrophages may not reach the cells latently infected with HIV-1 in other tissues. Although such an event is possible, studies have indicated the presence of HIV-1-infected cells in the vicinity of TB lesions. It has been shown that TB granulomas contain increased levels of HIV-1 Gag p24 and high reverse transcriptase activity compared to disease-free regions (9). Moreover, M. tuberculosis-triggered inflammation promotes the localization of HIV-infected cells at such inflammatory locations (4). Another limitation could be the lack of relevance of exosome-mediated HIV-1 activation in humans coinfected with HIV-M. tuberculosis; however, several previous studies suggested the significance of M. tuberculosis in modulating HIV-1 infection. For example, HIV-1-infected individuals are 20 to 30 times more likely to become infected with M. tuberculosis than uninfected individuals (88). Furthermore, HIV/*Mtb*-coinfected individuals exhibit lower overall survival rates despite antiretroviral therapy (ART) (1). Also, transient bursts of HIV-1 replication and replenishment of viral reservoir are evident even in patients whose viral load is well controlled by ART (89, 90). The mechanisms of viral bursts and renewal of viral reservoir are not entirely known. Since infection with M. tuberculosis is known to increase the levels of HIV-1 RNA in plasma of patients even in the presence of ART (91), we believe that mechanisms such as exosome-mediated HIV-1 reactivation may influence the size of latent virus reservoir. This could have adverse health-related outcomes in poorly managed or noncomplaint patients (92, 93). Taking the data together, our report provides initial observations from the study of M. tuberculosis exosome-mediated reactivation of HIV-1 and further work is needed to reveal the significance and complete mechanism of reactivation *in vivo*.

## MATERIALS AND METHODS

### Cell lines and bacterial culture.

Human monocytic cell line U937, murine macrophage cell line RAW264.7, and the chronically HIV-1-infected U1 (monocytic) and J1.1 (T-lymphocytic) cell lines were cultured as described earlier (20, 94). J-Lat 10.6 cells (Jurkat T lymphocytes, representing a reporter cell line containing a full-length integrated HIV-1 genome with a nonfunctional *env* gene whose lack of functionality was due to a frameshift and to the presence of a *gfp* gene in place of the *nef* gene) were maintained in RPMI 1640 (Cell Clone) supplemented with 10% heat-inactivated fetal bovine serum (FBS) (Sigma-Aldrich), 2 mM l-glutamine, 100 U/ml penicillin, and 100 mg/ml streptomycin (Sigma-Aldrich) at 37°C and 5% CO_2_. The bacterial strains used in this study were wild-type Mycobacterium tuberculosis H37Rv, Mycobacterium bovis bacillus Calmette-Guérin (BCG), single-drug-resistant (SDR) clinical isolate BND-320, multidrug-resistant (MDR) clinical isolates Jal-1934 and Jal-2261, extensively drug-resistant (XDR) clinical isolate MYC431 (kind gift from Kanury V.S. Rao, International Centre for Genetic Engineering and Biotechnology [ICGEB], New Delhi), and the *MtbΔRD1* mutant (kind gift from David Sherman, University of Washington, School of Medicine, Seattle, WA). The strains were grown to mid-log phase (optical density at 600 nm [OD_600_] of 0.8) as described previously (94). For tdTomato-expressing M. tuberculosis strains, competent cells were prepared as described previously (94) and were electroporated using 1 μg of the pTEC27 plasmid (pMSP12::tdTomato; kind gift from Deepak Saini, Indian Institute of Science [IISc], Bangalore, India) with settings of 2.5 kV voltage, 25 μF capacitance, and 1,000 Ω resistance in a Bio-Rad Gene Pulser. Electroporated bacilli were kept for overnight recovery followed by selection on 7H11 agar plates containing hygromycin (50 μg/ml). After 21 days of selection, bacteria were grown in 7H9 broth to mid-log phase and used for further studies. For generating heat-killed M. tuberculosis (Hk-*Mtb*), bacilli were killed by resuspending the pellet in 2 ml of RPMI 1640 and heating it to 80°C for 30 min (95).

### M. tuberculosis labeling with PKH26 GL, complement opsonization, and infection.

Freshly grown M. tuberculosis bacilli were labeled with fluorescent lipophilic dye PKH26 GL (Sigma-Aldrich) per the manufacturer’s instructions to prepare red-labeled bacteria to distinguish between M. tuberculosis-infected and bystander cells. Fluorescent staining was performed at a final concentration of 10 μM for 15 min at room temperature, and FBS was added to terminate the labeling process. Bacilli were then washed thoroughly with phosphate-buffered saline (PBS) and resuspended in RPMI 1640. For complement opsonization, M. tuberculosis bacilli were incubated in 50% human serum for 30 min at 37°C (96) and then washed with PBS. For infection, U937 cells stably expressing Grx1-roGFP2 in the cytosol were seeded at a density of 0.2 million per well in 24-well plates with 5 ng/ml of phorbol 12-myristate 13-acetate (PMA; Sigma-Aldrich Co., Saint Louis, MO, USA) for 24 h. Cells were rested overnight following chemical differentiation to ensure that they had reverted to a resting phenotype before infection. Differentiated U937 cells were then infected with M. tuberculosis at an MOI of 10 and incubated for 4 h at 37°C in 5% CO_2_. Cells were then treated with amikacin at 200 μg/ml for 2 h to kill extracellular bacteria. After infection, cells were washed thoroughly with PBS and resuspended in complete media (RPMI 1640 supplemented with 10% FBS).

### Redox potential measurements.

Intracellular redox potential measurements were performed as described previously (20). Briefly, U937 cells stably expressing Grx1-roGFP2 in the cytosol were infected with PKH26-labeled M. tuberculosis. At indicated time points, cells were treated with 10 mM N-ethylmaleimide (NEM) for 5 min and fixed with 4% paraformaldehyde (PFA) for 15 min at room temperature. After washing with PBS, cells were analyzed using a FACSVerse flow cytometer (BD Biosciences). The biosensor response was measured by analyzing the ratio upon excitation at 405 and 488 nm with fixed emission at 510 nm. Data were analyzed using FACSuite software. For each experiment, the minimal and maximal fluorescence ratios, which correspond to 100% sensor reduction and 100% sensor oxidation, respectively, were determined. Cumene hydroperoxide (CHP; 0.5 mM) was used as the oxidant and dithiothreitol (DTT; 40 mM) as the reductant. *E_GSH_* levels were measured using the Nernst equation as described previously (20).

### Coculture of U937 macrophages with J-Lat and U1 cells.

Cocultures were grown according to established protocols (97). U937 cells were seeded at a density of 0.2 million/ml in 24-well plates and were infected with M. tuberculosis as described earlier (97). J-Lat and U1 cells were added at a density of 0.1 million/ml on uninfected or infected U937 monolayers after amikacin treatment. Cocultures were grown in complete medium (RPMI 1640 plus 10% FBS, 2 mM l-glutamine) at 37°C and 5% CO_2_ for 5 days in the case of J-Lat cells and 48 h in the case of U1 cells. Fresh medium was added to J-Lat cells after 48 h. At indicated time points, the supernatants containing J-Lat and U1 cells were collected and centrifuged at 1,500 rpm for 5 min to harvest cells. J-Lat and U1 cells were fixed in 4% PFA for 15 min, centrifuged at 1,500 rpm for 5 min, and resuspended in PBS for flow cytometric analysis. PMA and TNF-α were used at final concentrations of 5 ng/ml and 10 ng/ml, respectively.

### Culturing of J-Lat and U1 cells in U937-conditioned media.

J-Lat and U1 cells were seeded at a density of 0.1 million/ml in 24-well plates in the absence or presence of a 2-fold dilution of the supernatants derived from uninfected or M. tuberculosis-infected U937 macrophages. J-Lat and U1 cells were also cultured in 2-fold dilutions of the culture supernatant collected from M. tuberculosis*-*infected U937 macrophages grown in the presence of 10 μM GW4869 inhibitor. The presence of intact M. tuberculosis cells in the supernatant was ruled out by passing the supernatant through a 0.2-μm-pore-size filter followed by plating of the supernatant on solid bacterial growth media. J-Lat and U1 cells were harvested at indicated time points and fixed in 4% PFA for 15 min. Cells were resuspended in PBS for flow cytometric analysis.

### HIV-1 p24 staining.

For intracellular p24 staining, U1 cells were washed with PBS containing 10% human serum followed by fixation and permeabilization using a fixation/permeabilization kit (eBiosciences). Permeabilized cells were then incubated with 100 μl of a 1:100 dilution of phycoerythrin (PE)-conjugated mouse anti-p24 monoclonal antibody (MAb) (KC57-RD1; Beckman Coulter, Inc.) for 30 min at 4°C with intermittent mixing. After the samples were washed twice, the cells were analyzed by flow cytometry.

### qRT-PCR.

Total RNA was isolated using an RNeasy minikit (Qiagen), according to the manufacturer’s instructions. RNA (500 ng) was reverse transcribed to cDNA using an iScript cDNA synthesis kit (Bio-Rad) and random oligonucleotide primers. p24-specific primers (forward, 5′-ATAATCCACCTATCCCAGTAGGAGAAAT-3′; reverse, 5′-TTGGTTCCTTGTCTTATGTCCAGAATGC-3′) were used to perform PCR. Gene expression was analyzed using real-time PCR, iQTM SYBR green Supermix (Bio-Rad), and a CFX96 RT-PCR system (Bio-Rad). Data analysis was performed with CFXManager software (Bio-Rad). The expression level of each gene was normalized to that of the human β-actin gene (forward, 5’ATGTGGCCGAGGACTTTGATT-3′; reverse, 5′-AGTGGGGTGGCTTTTAGGATG-3′).

### Isolation and purification of exosomes.

Exosomes were isolated and purified using ultrafiltration and exosome precipitation techniques as described previously (98, 99). Briefly, 60 million RAW264.7 macrophages (6 million cells per 100-mm-diameter cell culture dish) were cultured in cell culture dishes as described before (94) followed by infection at an MOI of 10 with M. tuberculosis, Hk-*Mtb*, Jal-1934 (MDR), or MYC431 (XDR) or were left uninfected. After infection, cells were washed thoroughly with PBS and incubated in serum-free Dulbecco’s modified Eagle’s medium (DMEM) (Cell Clone) at 37°C and 5% CO_2_. Serum-free medium was used to avoid contamination with exosomes present in the FBS. Culture supernatants were collected at 24 h p.i. and centrifuged at 5,000 rpm for 15 min at 4°C to remove cells, cell debris, or any M. tuberculosis present in the supernatant. Cleared culture supernatants were filtered twice through 0.2-μm-pore-size filters. Exosomes are 30 to 100 nm in diameter and filter freely through 0.2-μm-pore-size filters. Filtered supernatants were concentrated to 1 ml using an Amicon Ultra-15 filter (Merck) with a 100-kDa molecular weight cutoff (MWCO) in a swing-out rotor (Thermo Scientific SL 16) at 4°C and 4,000 × *g*. An equal volume of ExoQuick Ultra reagent (Systems BioSciences Inc., CA), which is the most advanced reagent used to isolate exosomes in pure form, was added to concentrated culture supernatant, and the resulting solution was mixed by inverting the tube and allowing it to stand overnight at 4°C. This mixture was then centrifuged at 1,500 × *g* for 30 min. The supernatant was discarded, and the pellet, which consisted of exosomes, was then resuspended in sterile PBS mixed with protease inhibitor cocktail (Pierce Thermo Fisher Scientific) and stored at −80°C until further analysis.

### Mouse infection and isolation of exosomes from serum and lungs.

BALB/c mice (6 to 8 weeks old) were infected with M. tuberculosis 100 bacilli per lung by aerosol or left uninfected as described previously (100). At 20 weeks p.i., animals were euthanized and serum and lungs were collected. Exosomes were isolated from mouse serum by precipitation in ExoQuick Ultra solution overnight per the manufacturer’s instructions.

To isolate exosomes from lungs, 2 ml of tissue digestion mix (serum-free RPMI 1640 with 200 μg/ml Liberase DL [Sigma-Aldrich] and 100 μg/ml of DNase [Thermo Fisher Scientific]) was added to one whole lung and transferred to C-tubes. Lungs were homogenized on a gentleMACS Dissociator (Miltenyi Biotec). Samples were incubated for 30 min at 37°C at 70 to 100 rpm followed by further homogenization. Lung homogenates were passed through a 40-μm-pore-size cell strainer and centrifuged at 1,500 rpm for 5 min. Supernatants were collected and passed through a 0.22-μm-pore-size filter. Filtered supernatants were concentrated to 1 ml, and exosomes were isolated using ExoQuick Ultra precipitation solution as described above. Exosomes were stored at −80°C for future analysis.

### Virus production.

HIV-1 production was carried out using Lipofectamine 2000 transfection reagent (Invitrogen, Life Technologies), according to the manufacturer’s protocol, in HEK293T cells with HIV-1 NL4-3 infectious molecular clone (NIH AIDS Reagent Program, Division of AIDS, NIAID, NIH). The medium was changed 6 h posttransfection, and supernatants were collected after 60 h, centrifuged (5 min, 1,500 rpm, room temperature), and filtered through a 0.45-μm-pore-size membrane filter (MDI; Membrane Technologies) to clear cell debris. Virus was concentrated using 5× Retro-Concentin (System Biosciences) as per the manufacturer’s protocol, and the virus pellet obtained was divided into aliquots in Opti-MEM and stored at −80°C. Virus concentrations were estimated by p24 titration using HIV-1 p24 ELISA (J. Mitra and Co. Pvt. Ltd., India) according to the manufacturer’s instructions.

### Isolation and HIV-1 infection of primary T cells.

Peripheral blood mononuclear cells (PBMCs) were isolated using Ficoll-Paque-based density gradient centrifugation from blood samples of healthy donors, donated after informed consent, approved by the IISc Ethical Committee. Primary CD4^+^ T cells were purified from PBMCs using an EasySep human CD4^+^ T cell isolation kit (StemCell Technologies, Canada). Purified resting CD4^+^ T cells were cultured for 3 days after isolation at 37°C in 5% CO_2_ in complete media containing RPMI 1640 supplemented with 10% FBS, 100 U/ml interleukin-2 (IL-2) (Peprotech, London, United Kingdom) (specific activity, 10 U/ng), and 1 μg/ml phytohemagglutinin (PHA) mitogen (Thermo Fisher Scientific). Subsequently, 250,000 activated primary CD4^+^ T cells were seeded per well in a 96-well flat-bottom plate in complete media containing 100 U/ml IL-2. HIV-1 (100 ng p24) was added per well in a final volume of 200 μl. Cells were subsequently subjected to spinoculation at 1,000 × *g* for 90 min at 32°C. Cells were then washed and incubated in complete media containing 100 U/ml IL-2 and treated with 100 μg/ml of purified exosomes isolated from live M. tuberculosis*-*infected, Hk-*Mtb-*infected, and M. tuberculosis Δ*RD1-*infected RAW264.7 macrophages or left untreated. On day 5 after HIV-1 infection, virus replication was quantified by estimating virion release in the extracellular media. Briefly, supernatant was harvested from infected cells and centrifuged at 400 × *g* for 10 min, and virus concentrations were estimated by HIV-1 p24 ELISA.

### Western blotting.

Exosomes were lysed in radioimmunoprecipitation assay (RIPA) buffer (25 mM Tris-HCl [pH 7.6], 150 mM NaCl, 1% NP-40, 1% sodium deoxycholate, 0.1% SDS) with protease inhibitor (Pierce Thermo Fisher Scientific). Protein estimation was performed using a micro-bicinchoninic acid (micro-BCA) assay (Pierce Thermo Fisher Scientific). Unless specified otherwise, a 50-μg volume of protein was mixed with Laemmli buffer and heated at 95°C for 5 min followed by chilling on ice for 5 min before loading onto an SDS-PAGE gel was performed. Western blotting was performed using LAMP2 (ab25631), Rab5b (BD-610281), Alix (CST-2171), CD63 (sc-15363), p24 (ab9071), and α-tubulin (CST-2144) as primary antibodies and goat anti-rabbit IgG HRP (CST-7074) and horse anti-mouse IgG (CST-7076) as secondary antibodies.

### Transmission electron microscopy.

Exosomes were fixed in 4% PFA for 10 min, and 10 μl of sample was mounted onto a carbon Formvar-coated copper grid. The samples were allowed to adsorb on grids for 10 min to form a monolayer, and the remaining sample was wiped off using clean filter paper. Grids were washed thrice with PBS followed by incubation in a 50-μl drop of 1% glutaraldehyde for 5 min. Grids were washed thoroughly with PBS and stained with 2 μl of filtered 2% uranyl acetate solution for 1 min. After three washes with PBS, grids were dried at room temperature.

For immunogold labeling of exosomes with anti-CD63 antibody, exosomes were fixed in 4% PFA. Fixed exosome samples were mounted on carbon Formvar-coated 300-mesh copper grids for 10 min before wiping off the excess samples with the help of filter paper. Grids were blocked in 0.5% bovine serum albumin (BSA)–PBS (blocking buffer) for 30 min and washed thrice in PBS. Grids were then incubated in blocking buffer (negative control) or primary antibody (CD63) diluted to 1:100 for 1 h. Grids were washed thoroughly with PBS followed by incubation with anti-rabbit 10-nm-diameter gold antibody (ab27234) diluted to 1:250 for 1 h. Grids were then incubated in 1% glutaraldehyde for 5 min to fix the immunoreaction. Negative staining was performed using 2% aqueous uranyl acetate solution for 1 min. After washing was performed, grids were air dried and viewed with a JEM 1011 transmission electron microscope at 120 kV.

### NanoString gene array.

U1 cells were treated with a 100 μg/ml concentration of exosomes for 12 h, and total RNA was isolated using an RNeasy minikit (Qiagen) according to the manufacturer’s instructions. RNA concentration and purity were measured using a Nanodrop spectrophotometer (Thermo Fisher Scientific, Waltham, MA). An nCounter gene expression assay was performed according to the manufacturer’s protocol. The assay utilized a custom-made NanoString codeset designed to measure 185 transcripts, including 6 putative housekeeping transcripts (see Table S1a in the supplemental material). This custom-made panel included genes reported to be differentially regulated in response to HIV infection and oxidative stress. The data were normalized to the average counts for all housekeeping genes in each sample and analyzed with nSolver software (NanoString Technologies).

### OCR measurement.

Oxygen consumption rates (OCR) were measured at 37°C using a Seahorse XFp extracellular flux analyzer (Seahorse Bioscience). XF cell culture microplate plates were coated with 10 μl Cell-Tak (Sigma-Aldrich) reagent according to the manufacturer’s protocol. U1 cells were treated with exosomes isolated from M. tuberculosis-infected, Hk-*Mtb*-infected, or uninfected RAW264.7 macrophages at 100 μg/ml for 48 h. After 48 h, U1 cells were washed and seeded in a Seahorse flux analyzer microplate precoated with Cell-Tak at a density of 50,000 cells per well to generate a confluent monolayer of cells. An Agilent seahorse XFp Cell Mito Stress kit (Agilent Technologies) was utilized to carry out a mitochondrial respiration assay. Briefly, three OCR measurements were performed in XF assay media to measure basal respiration without addition of any inhibitor, followed by sequential exposure of cells to oligomycin (1 μM) and an ATP synthase inhibitor and three OCR measurements to determine ATP-linked OCR and proton leakage. Then, cyanide-4-(trifluoromethoxy)phenylhydrazone (FCCP; 0.25 μM), an electron transport chain (ETC) uncoupler, was injected to determine the maximal respiration rate and the spare respiratory capacity (SRC). Finally, antimycin A and rotenone (0.5 μM each), inhibitors of complex III and I, respectively, were injected to completely shut down the ETC to analyze nonmitochondrial respiration. Wave Desktop 2.6 software (Agilent) was used for the calculation of the parameters from the mitochondrial respiration assay. Data were normalized according to a previously described protocol (101).

### Proteomic analysis of exosomes by LC-MS/MS.

Proteins were extracted from exosomes for the immunoblotting experiment as described previously. Protein samples (30 μg) were resolved on 10% SDS-PAGE gel up to a distance of 3 cm and were stained with Coomassie brilliant blue R250. The lanes were cut into three equally sized bands. These bands were first reduced by the use of 5 mM Tris (2-carboxyethyl) phosphine hydrochloride (TCEP; Sigma-Aldrich) followed by alkylation with 50 mM iodoacetamide and were digested with 1 μg trypsin for as long as 16 h at 37°C. The digests were cleaned up using a C_18_ silica cartridge (The Nest Group, Southborough, MA), dried using a SpeedVac, and then resuspended in buffer A (5% acetonitrile–0.1% formic acid).

An EASY-nLC 1000 system (Thermo Fisher Scientific) coupled to a QExactive mass spectrometer (Thermo Fisher Scientific) fitted with nanoelectrospray ion source was used to perform LC-MS/MS. A 15-cm-long PicoFrit column filled with 1.8 μm of C_18_ resin (Dr. Maisch GmbH) was used to load and resolve 1 μg of the peptide mixture with buffer A. Loading and elution with a 0% to 40% gradient of buffer B (95% acetonitrile–0.1% formic acid) were performed at a flow rate of 300 nl/min at room temperature for 105 min. The MS was driven at a full-scan resolution of 70,000 at an *m*/*z* of 400, and the MS/MS scans were acquired at a resolution of 17,500 at an *m*/*z* of 400 using Top10 higher-energy collisional dissociation (HCD) data-dependent acquisition mode. Polydimethylcyclosiloxane (PCM) ions (*m*/*z* = 445.120025) were set up as the lock mass option for internal recalibration during the run. Determinations of MS data were carried out using a data-dependent Top10 method, which effectively chooses the most abundant precursor ions from a survey scan.

Raw files were analyzed using Thermo Proteome Discoverer 2.2 searched against the UniProt Mus musculus reference proteome database with both the PSM (peptide spectrum match) and protein FDR (false-discovery rate) values set to 0.01 using the percolator node. For Sequest HT searches, the precursor and fragment mass tolerances were set at 10 ppm and 0.5 Da, respectively. Protein quantification was done using the Minora feature detector node with default settings and considering only data representing high PSM confidence.

### Data processing and analysis.

Differential analysis was performed on label-free quantification data using R packages ProstaR and DAPAR. The intensity values were subjected to log transformation followed by filtering of rows containing NA values of ≥5 in the data. Imputation was performed using the R package Mice. Limma-moderated *t* testing was used to identify differentially expressed proteins, and *P* values were adjusted using the BH method for comparisons between two groups. All results of analyses of proteins with fold change values of >2 or ≤2 and with *P* values of <0.01 were considered significant. Functional classifications of differentially expressed proteins with GO and KEGG signaling pathways from comparisons between two groups were analyzed using R package-clusterProfiler.

### Statistical analysis.

Statistical analyses were performed using GraphPad Prism software (version 6.0). Comparisons of multiple groups were made by using either one-way or two-way analysis of variance (ANOVA) with the Bonferroni test. Differences with a *P* value of <0.05 were considered significant.

### Ethics statement.

This study was carried out in strict accordance with the guidelines provided by the Committee for the Purpose of Control and Supervision of Experiments on Animals (CPCSEA), Government of India. The protocol was approved by the Animal Ethics Committee (AEC) of the Indian Institute of Science (CAF/Ethics/485/2016).
