# Supplementary figures and images for: Mycobacterium tuberculosis Reactivates HIV-1 via Exosome-Mediated Resetting of Cellular Redox Potential and Bioenergetics
Source: mBio. 2020 Mar 3;11(2):e03293-19. doi: 10.1128/mBio.03293-19 (PMC7064780; doi:10.1128/mBio.03293-19)

**A**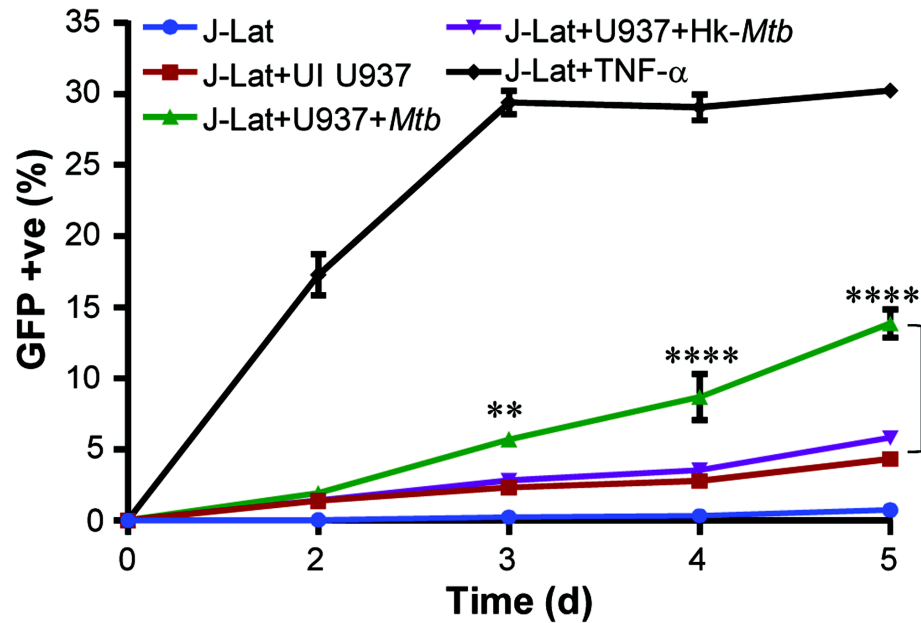**B**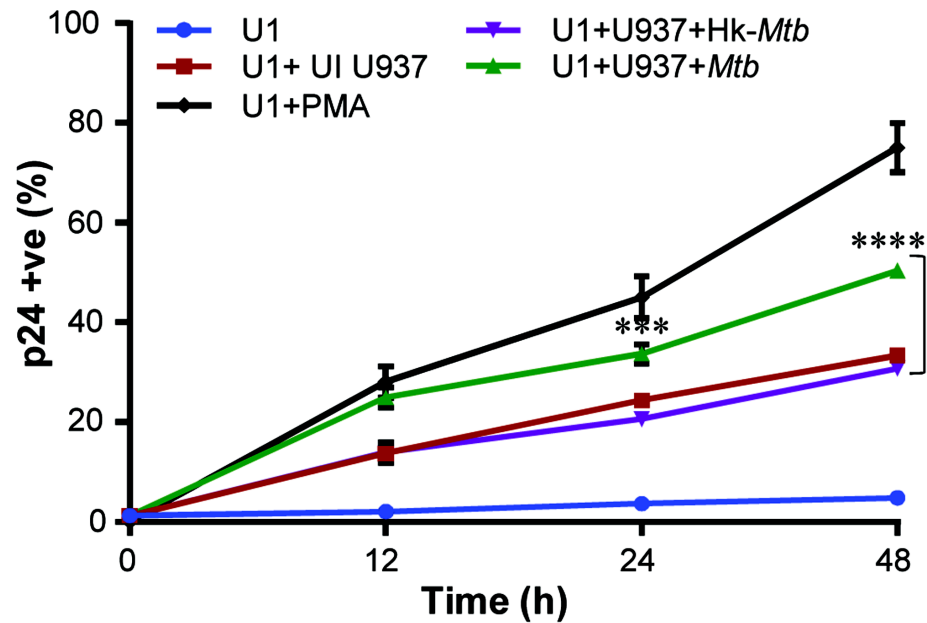

Supplement: FIG S1 [file mBio.03293-19-sf001.pdf]

**A**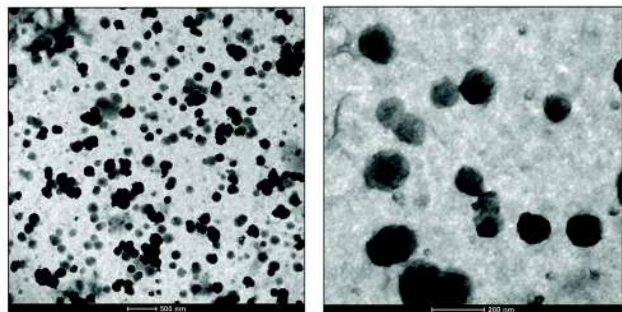**B**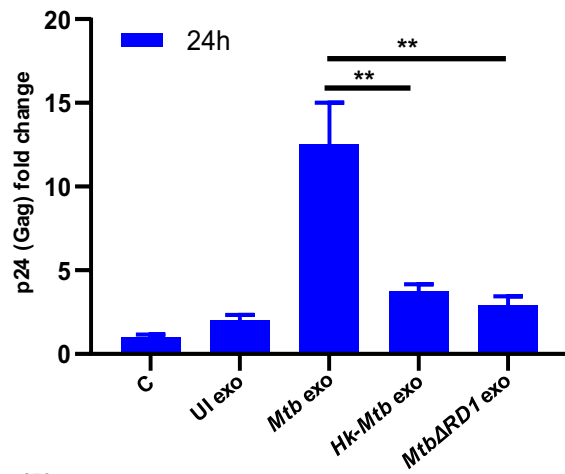**C**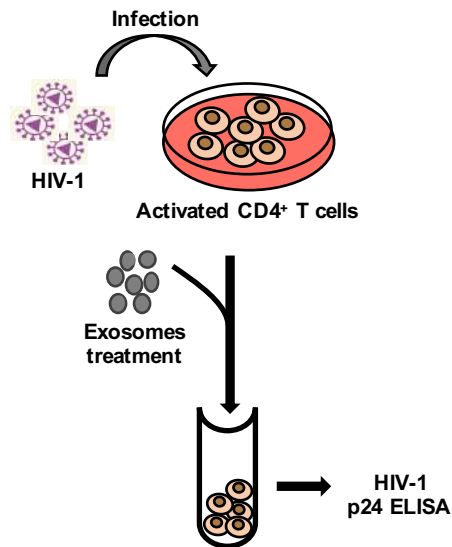**D**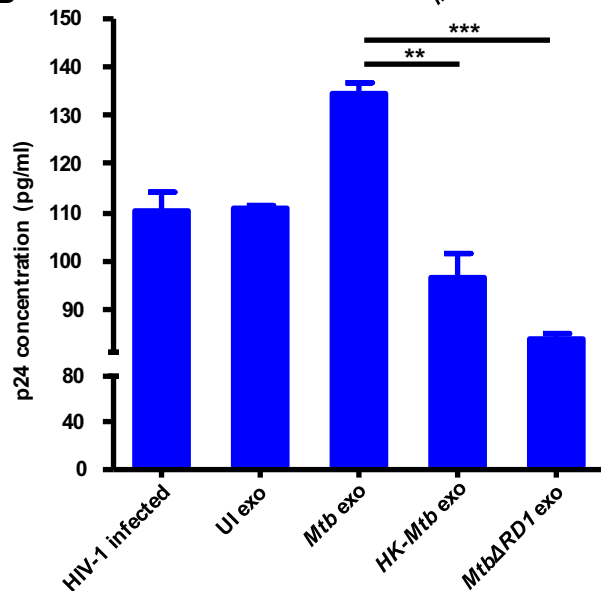

Supplement: FIG S2 [file mBio.03293-19-sf002.pdf]

**A**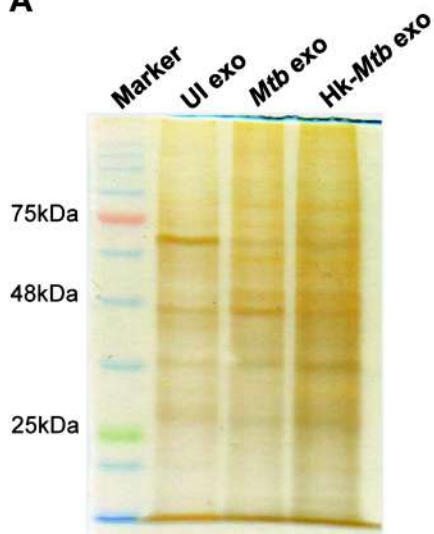**B**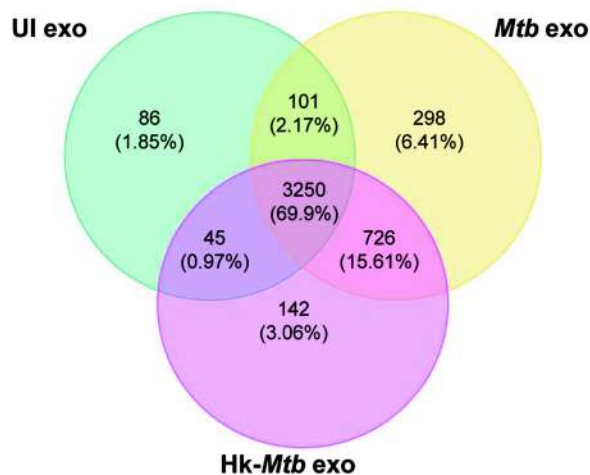**C**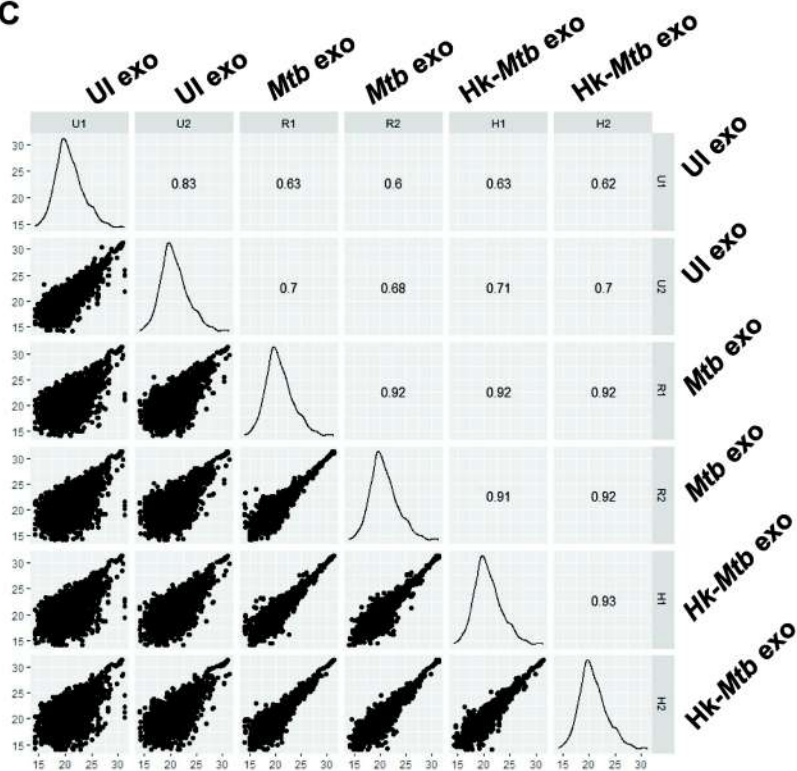

Supplement: FIG S3 [file mBio.03293-19-sf003.pdf]

A

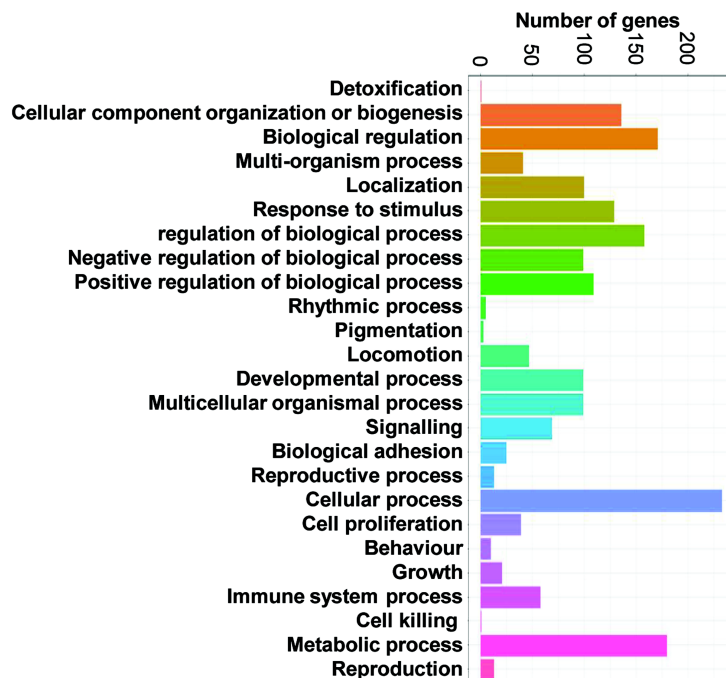

B

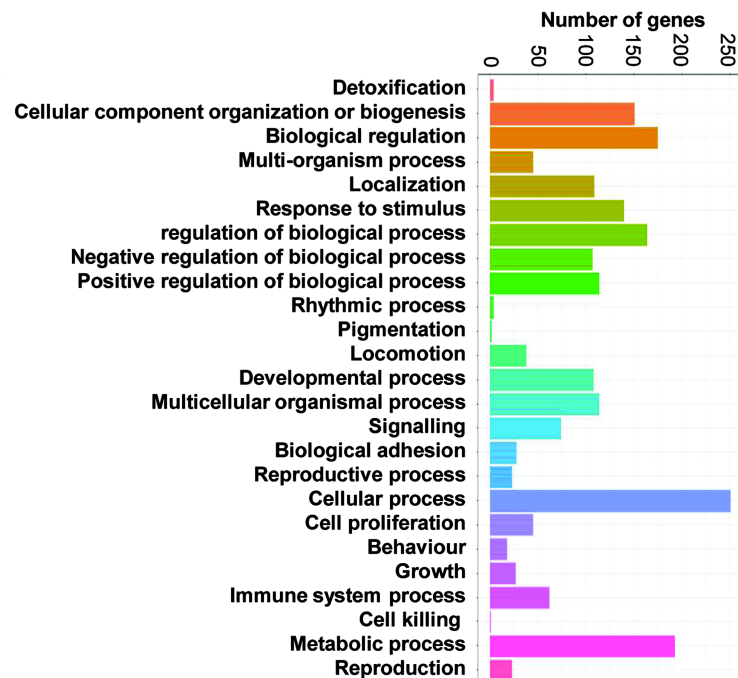

C

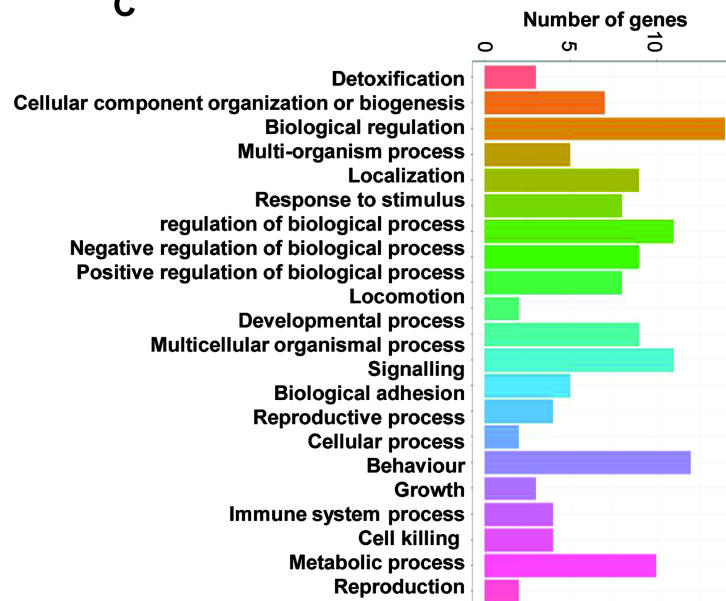

Supplement: FIG S4 [file mBio.03293-19-sf004.pdf]

**A**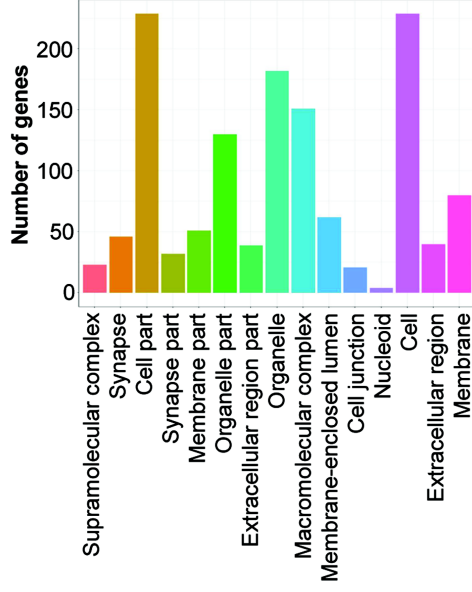**B**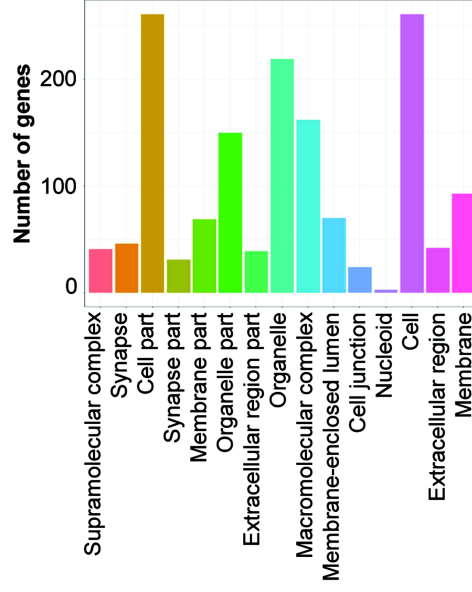**C**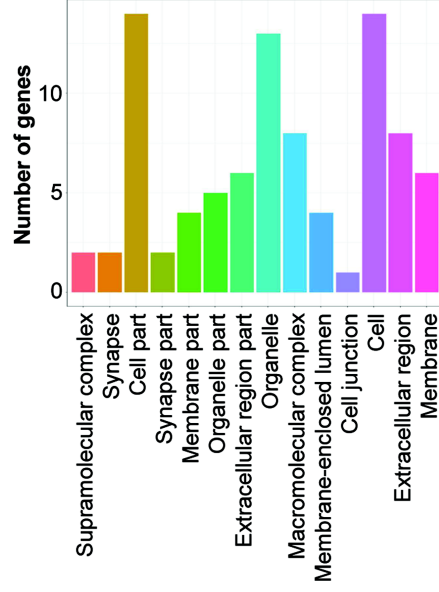**D**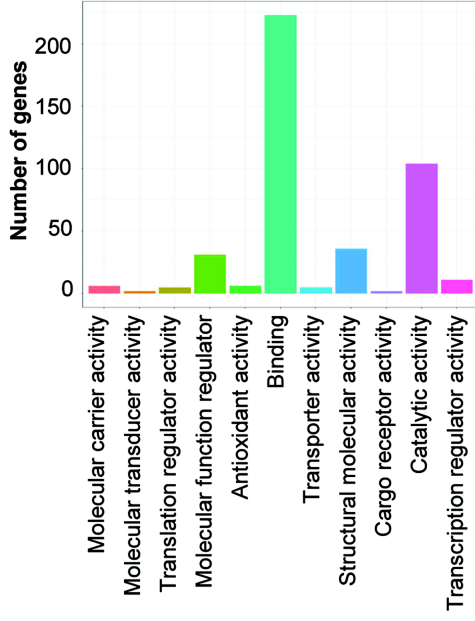**E**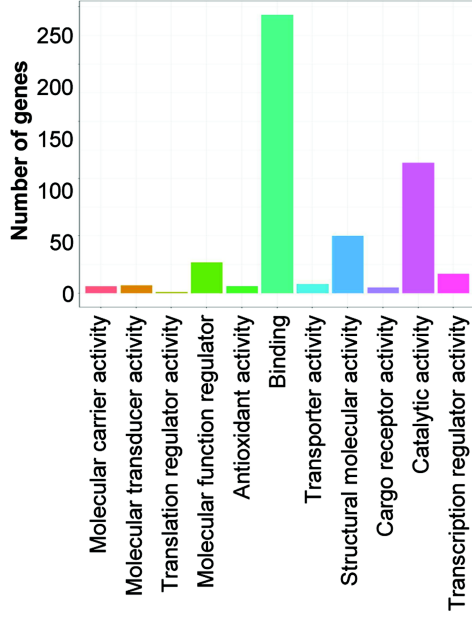**F**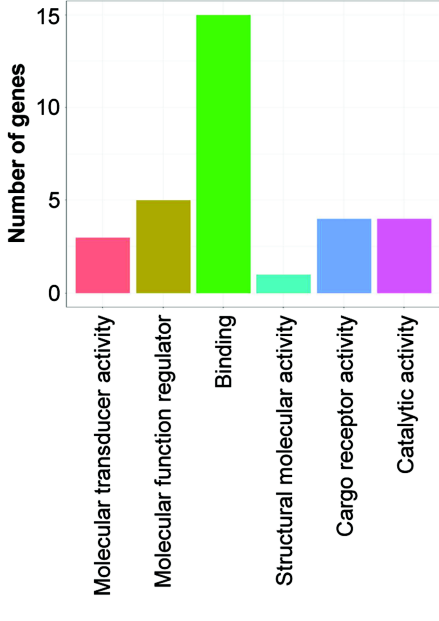

Supplement: FIG S5 [file mBio.03293-19-sf005.pdf]
